# Identification of Key Genes for the Ultrahigh Yield of Rice Using Dynamic Cross-tissue Network Analysis

**DOI:** 10.1016/j.gpb.2019.11.007

**Published:** 2020-07-28

**Authors:** Jihong Hu, Tao Zeng, Qiongmei Xia, Liyu Huang, Yesheng Zhang, Chuanchao Zhang, Yan Zeng, Hui Liu, Shilai Zhang, Guangfu Huang, Wenting Wan, Yi Ding, Fengyi Hu, Congdang Yang, Luonan Chen, Wen Wang

**Affiliations:** 1 State Key Laboratory of Genetic Resources and Evolution, Kunming Institute of Zoology, Chinese Academy of Sciences, Kunming 650223, China; 2 State Key Laboratory of Hybrid Rice, College of Life Sciences, Wuhan University, Wuhan 430072, China; 3 CAS Key Laboratory of Systems Biology, Center for Excellence in Molecular Cell Science, Institute of Biochemistry and Cell Biology, Shanghai Institutes for Biological Sciences, Chinese Academy of Sciences, Shanghai 200031, China; 4 Institute of Brain-Intelligence Technology, Zhangjiang Laboratory, Shanghai 201210, China; 5 Institute of Food Crop of Yunnan Academy of Agricultural Sciences, Kunming 650205, China; 6 School of Agriculture, Yunnan University, Kunming 650500, China; 7 BGI-Baoshan, Baoshan 678004, China; 8 Center for Ecological and Environmental Sciences, Northwestern Polytechnical University, Xi’an 710072, China; 9 School of Life Science and Technology, ShanghaiTech University, Shanghai 201210, China

**Keywords:** Dynamic cross-tissue (DCT), Systems biology, RNA-seq, Ultrahigh yield, Rice

## Abstract

Significantly increasing crop yield is a major and worldwide challenge for food supply and security. It is well-known that **rice** cultivated at Taoyuan in Yunnan of China can produce the highest yield worldwide. Yet, the gene regulatory mechanism underpinning this **ultrahigh yield** has been a mystery. Here, we systematically collected the transcriptome data for seven key tissues at different developmental stages using rice cultivated both at Taoyuan as the case group and at another regular rice planting place Jinghong as the control group. We identified the top 24 candidate high-yield genes with their network modules from these well-designed datasets by developing a novel computational **systems biology** method, *i.e.*, **dynamic cross-tissue (DCT)** network analysis. We used one of the candidate genes, *OsSPL4*, whose function was previously unknown, for gene editing experimental validation of the high yield, and confirmed that *OsSPL4* significantly affects panicle branching and increases the rice yield. This study, which included extensive field phenotyping, cross-tissue systems biology analyses, and functional validation, uncovered the key genes and gene regulatory networks underpinning the ultrahigh yield of rice. The DCT method could be applied to other plant or animal systems if different phenotypes under various environments with the common genome sequences of the examined sample. DCT can be downloaded from https://github.com/ztpub/DCT.

## Introduction

Utilization of the heterosis of hybrids was reported to increase the rice yield by 15%–25% during past decades in China [1]. Recently, based on the proportion of the national rice area represented by each location and rice cropping system, the national estimates of the potential rice yield in China are 6.8–9.8 metric tons per hectare (t·ha^−1^) whereas the farm yields range from 5.2 to 8.8 t·ha^−1^ [2]. However, rice breeding is now confronted with the challenge of overcoming the yield plateau [3]. Interestingly, Taoyuan of Yunnan in China is a well-known place where the highest rice yield in the world was recorded with an average rice yield of 13.91 t·ha^−1^ [3], [4]. Taoyuan is a dry and hot valley of the upstream part of Yangtze River in Yunnan Province, where the temperature difference is large after heading, and the humidity is slightly lower throughout the growing period. Consistently, during 4 years of experimentations at Taoyuan, we observed that the rice yield at Taoyuan is at least 70% higher than that obtained at Jinghong which is a control place located south of Yunnan Province with a similar environment to most rice planting areas under the same cultivation management. Therefore, some unidentified environmental differences could leave their imprint in the epigenome and modify gene expression and regulatory networks [3], [4]. However, the traditional differential expression analysis only compares the differential gene expression in one tissue, and ignores the gene networks in multiple tissues. To investigate the genes and gene regulatory networks driving such ultrahigh yield observed at Taoyuan, we developed a new dynamic network analysis across tissues and developmental stages to identify candidate genes/networks accounting for the ultrahigh yield.

**Figure 1:**
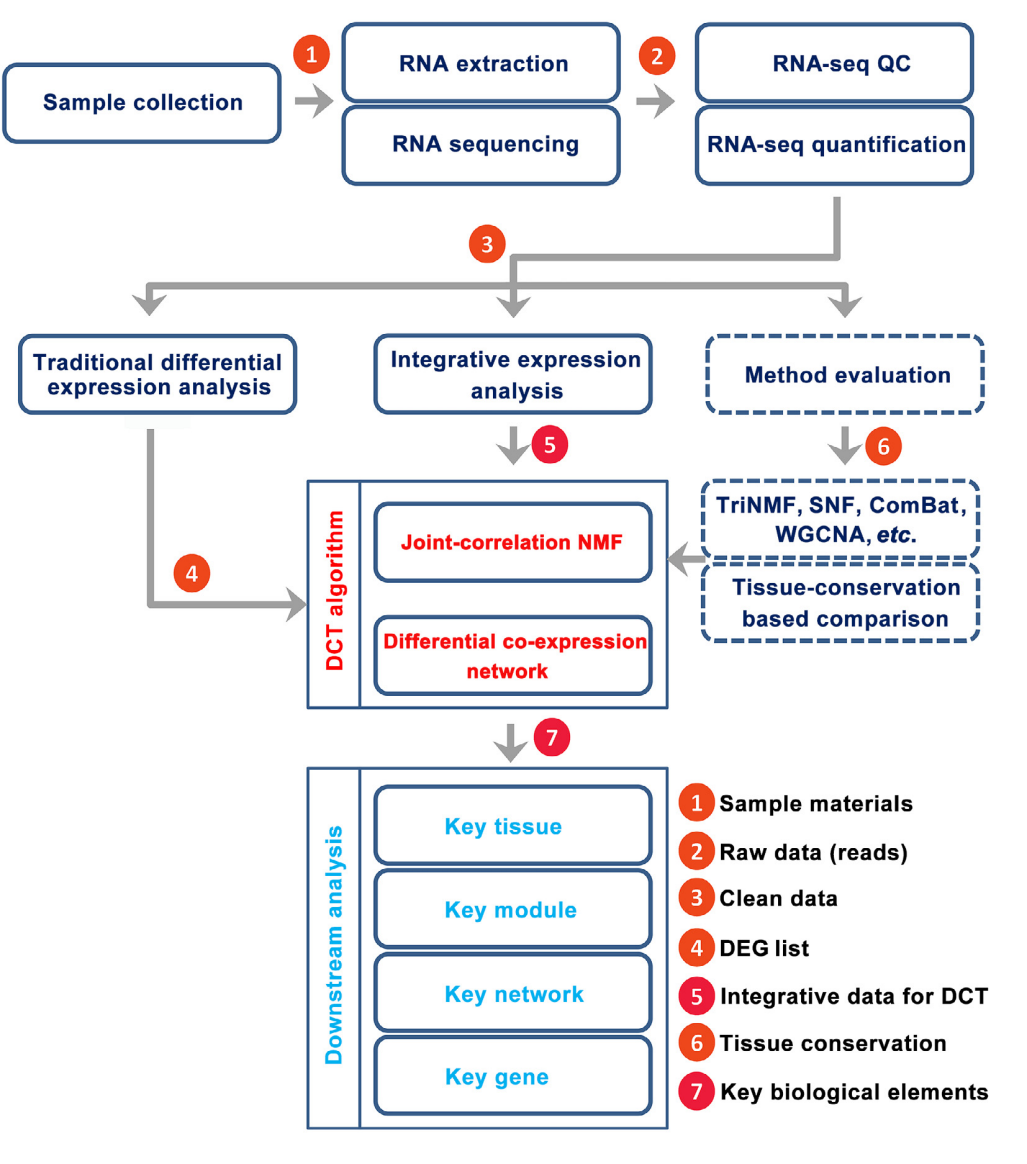
**Flow chart of the identification of key genes using DCT network analysis** Briefly, raw data from transcriptomic datasets of different tissues were obtained. After filtering, the clean data were analyzed by traditional differential expression analysis to identify DEGs. After removing any redundancies, feature genes (5746 genes in the present study), including the DEGs with a 1.2 fold change in expression level, were obtained for further analysis. The conservation levels of different tissues and their gene sets were then assessed, and the efficiency of different integration methods or models were evaluated. Finally, based on the jcNMF algorithm and DENs, the DCT network analysis approach was developed to capture the key genes, key gene modules and key gene network as well as the key tissue. DCT, dynamic cross-tissue; DEG, differentially expressed gene; NMF, non-negative matrix factorization; SNF, similarity network fusion; TriNMF, conventional NMF-based method; WGCNA, weighted correlation network analysis; QC, quality control.

Integration or meta-analysis is a recently developed approach to study biological multi-tissue transcriptome data [5], [6], [7], [8], [9]. Non-negative matrix factorization (NMF) is one such methodology, which in particular has the advantage to integrate multi-type high-throughput data, including RNA-seq or microarray data. Thus, NMF has been widely adopted in integration analysis involving heterogeneous data [10]. However, the conventional methods usually cannot take these constraints into consideration in a biological context, such as tissue types and developmental stages, which severely limits their effectiveness. To integrate gene expression data across tissues and developmental stages by directly exploiting the biological context, we developed a new computational systems biology method, *i.e.*, dynamic cross-tissue (DCT) network analysis. DCT is based on the newly proposed joint-correlation NMF (jcNMF) and differential co-expression networks (DENs) [11], [12]. Based on the integrative results of the jcNMF calculation, a systematic gene selection approach based on DENs was used to identify the key genes and the key gene modules of high yield with some functional validations (Figure 1). This comprehensive DCT analysis of multiple pairs of tissues across different developmental stages obtained from our field experiments provides a clear and inclusive view of the genes and networks driving the ultrahigh yield of rice at Taoyuan.

## Results

### Special environment and ultrahigh yield of rice at Taoyuan

**Supplementary Figure S1:**
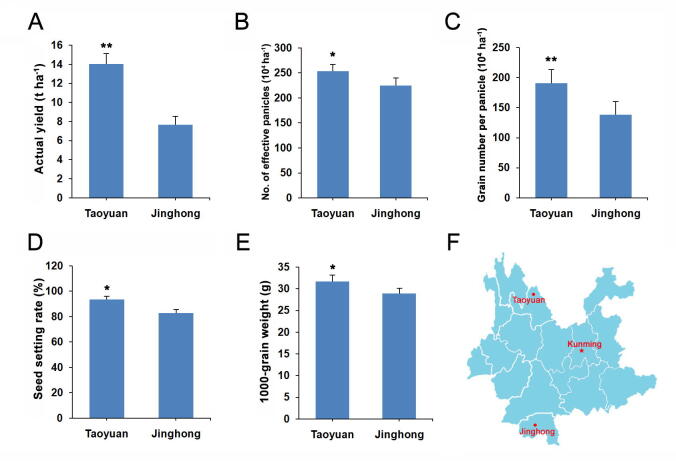
**Four years' average yield and yield components of the variety 9311 at Taoyuan and Jinghong**. **A.** Actual yield. **B.** Effective panicle numbers. **C.** Grain number per panicle. **D.** Seed setting rate. **E.** 1000-grain weight. *, *P* < 0.05; **, *P* < 0.01. **F.** Location of Taoyuan and Jinghong in Yunnan, China.

Field experiments using rice variety 9311 were conducted in 2010, 2011, 2013, and 2014 at Taoyuan and Jinghong, Yunnan, China (Figure S1 and Table 1). In the four testing years, the rice variety 9311 consistently showed significantly higher yields (88.91%, 74.60%, 92.61%, and 78.28% higher, respectively) at Taoyuan than that at Jinghong (Table 1 and Figure S1), whereas the yield at Jinghong (approximately 7 t·ha^−1^) was comparable to that at other typical *indica* rice planting areas [2], [13]. These results showed that we could consistently obtain an ultrahigh yield of rice at Taoyuan, which is much higher than the gain of hybrid rice with only a 15%–25% increase [3], [13].

**Table 1:** **Yield and yield components of rice variety 9311 at Taoyuan and Jinghong in 2010–2014**

*Note*: Significant differences between Taoyuan and Jinghong were determined by Student’s *t*-test. *, *P* < 0.05; **, *P* < 0.01. Number of grains per panicle includes the number of actual grains and the number of shrunken grains. Theoretical yield (t·ha^−1^) is calculated according to the formula: No. of effective panicles (10^4^ ha^−1^) **×** No. of grains per panicle × seed setting rate × 1000-grain weight **×** 10^−5^. Yield increase is calculated according to the formula: (actual yield_Taoyuan_ − actual yield_Jinghong_)/actual yield_Jinghong_.

Because Taoyuan is a dry and hot valley of the upstream part of Yangtze River in Yunnan Province, we selected Jinghong, which is a typical *indica* rice planting region, as the control place (Figure S1). We recorded the temperature, humidity and monthly rainfall. And the records showed that Taoyuan has a high temperature before heading, and a low temperature after heading, which results in a high temperature difference, whereas the humidity was slightly low throughout the growing period (Table S1). The rainfall exhibited the largest difference between the two places, but we had good irrigating systems to avoid drought in the plots. We strictly used the same crop management practice, including the same plot area, planting density and use of fertilized nitrogen (225 kg·N·ha^−1^) at the two places. We set up 3–4 replicates of 15 m^2^ plots and conducted careful phenotyping throughout the growth period.

**Figure 2:**
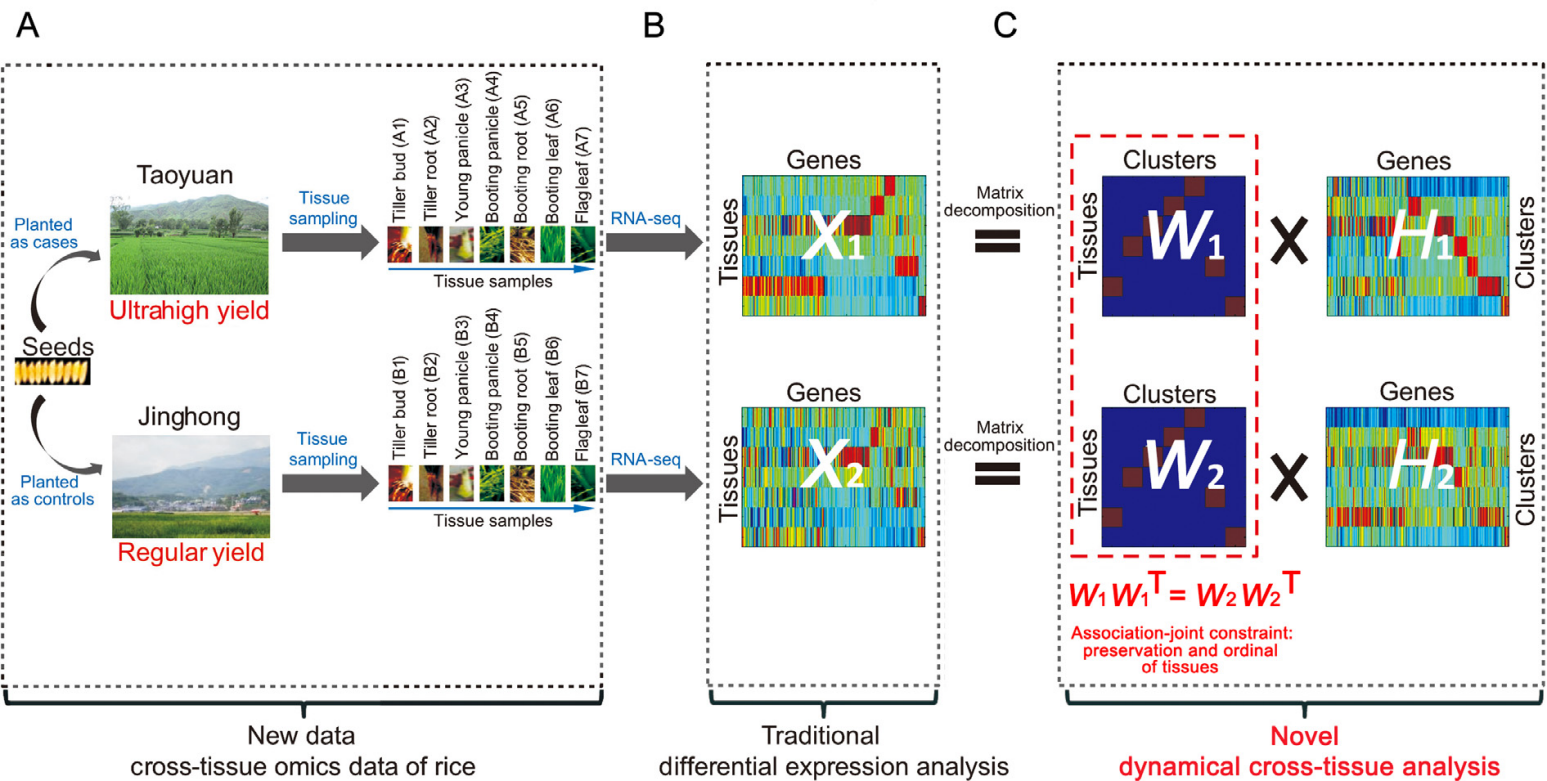
**Workflow of the DCT network analysis** The diagrammatic sketch compares the process of DCT analysis with traditional differential expression analysis. *X* denotes the non-negative dataset matrix of Taoyuan or Jinghong, *W* and *H* are two factor matrices of *X*. *W* is the gene-modules (or networks) of the samples (or individuals) (*i.e.*, the developmental gene co-expression patterns among rice tissues), and H represents the gene-module expressions among samples corresponding to phenotypes (*i.e.*, the tissue-specific gene-module levels of each rice tissue). **A.** RNA-seq data of different tissues from Taoyuan (case) and Jinghong (control). A1−A7, the corresponding seven tissues from Taoyuan, and B1−B7, the corresponding seven tissues from Jinghong, which represent sample label in our deposited data. **B.** Traditional differential expression analysis and gene expression comparison of Taoyuan (*X*_1_) and Jinghong (*X*_2_). **C.** Our new DCT analysis. Using jcNMF, we factorize *X* into *W* (*W*_1_, Taoyuan; *W*_2_, Jinghong) and *H* (*H*_1_, Taoyuan; *H*_2_, Jinghong). The conserved tissue correlations *W*_1_*W*_1_^T^ = *W*_2_*W*_2_^T^, rather than the conserved gene module compositions *W*_1_ = *W*_2_ in the case and control were used. T represents the matrix transpose. High-yield candidate genes were identified by jointly analyzing the DENs (see more details in Figure 4 and in the Methods and Supplementary material). DEN, differential co-expression network; jcNMF, joint-correlation non-negative matrix factorization.

**Figure 3:**
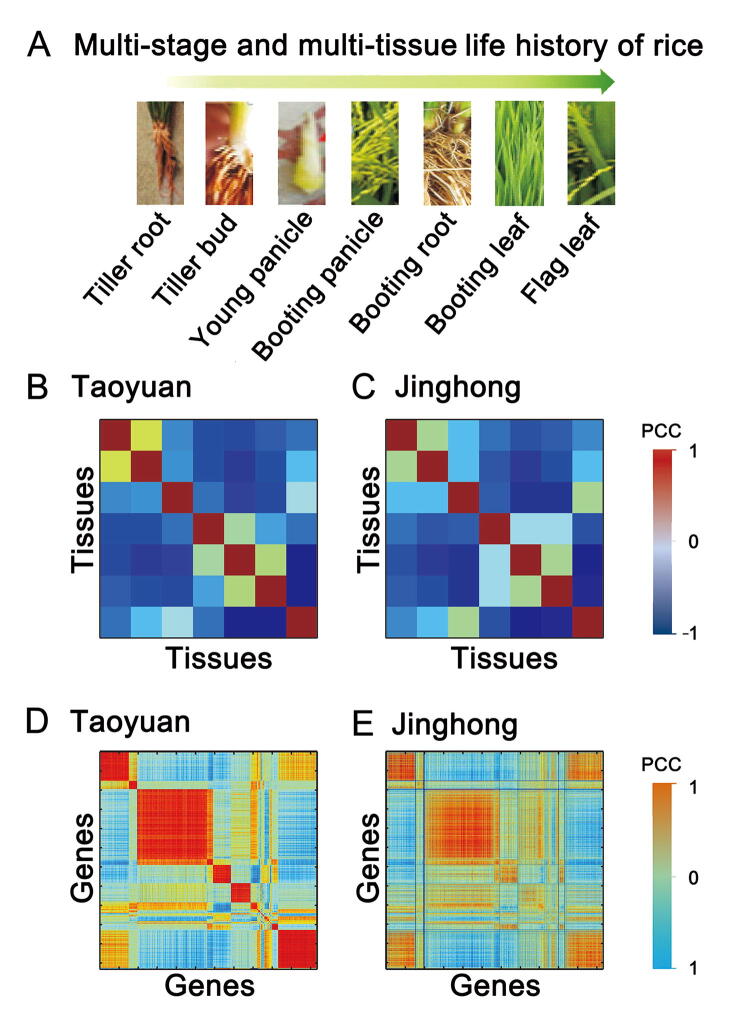
**Evaluation of the conservation levels of tissues and genes between Taoyuan and Jinghong samples** **A.** Developmental order of tissues sampled in this study. **B.** Tissue clustering analysis based on PCC for Taoyuan samples. **C.** Tissue clustering analysis based on PCC for Jinghong samples. **D.** Feature gene clustering analysis based on PCC for Taoyuan samples. **E.** Feature gene clustering analysis based on PCC for Jinghong samples. The colors from blue to dark red represent the increase in the correlation coefficient in both tissue and gene analysis. PCC, Pearson’s correlation coefficient.

We carefully dissected the phenotypic differences that might have contributed to the ultrahigh yield, and found that the number of effective panicles, grain numbers per panicles, seed setting rate, and 1000-grain weight all contributed to the ultrahigh yield at Taoyuan (Figure S1 A–E). However, none of the traits showed > 70% increases at Taoyuan compared with those at Jinghong. This finding suggests that the ultrahigh yield observed at Taoyuan is a collective result from these traits combined with the underlying gene regulation and indicates that a systematic approach is needed to dissect such a complex trait. Because these four traits are related to tillering, panicle development, and photosynthesis of flag leaves at the grain filling stage, we collected transcriptome data from the tiller bud, tiller root, young panicle, booting panicle, booting leaf, booting root, and flag leaf from Taoyuan and Jinghong rice, respectively. This study aimed to reveal the internal gene regulatory mechanisms accounting for the ultrahigh yield detected at Taoyuan (Figure 2 and Figure 3A).

### Identification of candidate high-yield genes through DCT meta-analysis of transcriptomic data

To systematically identify the key genes across multiple tissues for ultrahigh yield observed at Taoyuan, we developed a novel algorithm (DCT) differing from the traditional expression analysis (Figure 1, Files S1 and S2, https://github.com/ztpub/DCT). The mathematical model used in the DCT analysis utilizes joint correlation information (*i.e.*, soft constraints on tissue correlations in terms of gene modules) of NMF (jcNMF) instead of the conventional joint value (*i.e.*, hard constraints on tissue compositions in terms of gene modules) of NMF [9]. We showed that the joint correlation in jcNMF can well characterize the associations among tissues from the observed data between the case and control (Figure 3B and C). Furthermore, based on the results from the jcNMF calculation, a systematic gene selection approach based on DENs [11] was used to capture the key genes and the key gene modules of high yield with some validations from the additional field and functional experiments.

The DCT approach maps genotype to phenotype via gene networks (or modules), *i.e.*, genotypes → networks → phenotypes, rather than via directly linking/bridging the genotype and phenotype, *i.e.*, genotypes → phenotypes in the traditional way. There are 42,145 genes in the rice genome (IRGSP-1.0, http://rapdb.dna.affrc.go.jp/) and we obtained transcriptome datasets from 7 tissues collected in our field experiments. Theoretically, we would obtain a non-negative matrix *X* of 7 × 42,125 that includes all the raw data from either the case or the control. Using traditional differential expression analysis, a total of 343 differentially expressed genes (DEGs) was identified (Table S2). Therefore, we excluded 42,125 genes without significant expression changes (based on 1.2 fold change on the expression level as the cutoff) between the case and control samples, and thus, 4714 DEGs were included in *X*. Experientially, the threshold of fold change used is 1.2, as a conventional two fold change will be too strict, which can permit more moderate (candidate) DEGs (usually including important transcriptional factors (TFs)) to be considered in the downstream analysis (*i.e.*, network-based analysis). Because TFs play important roles in gene regulation, we kept 1251 rice TFs from PlantTFDB in the analysis without considering their expression changes [14]. In addition, 26 genes which have been reported to be related to rice yield until now (Dec 16, 2014) were included in the matrix regardless of their expression changes. These 26 genes were identified by other research groups using map-cloning, and their functions in rice yield were very clear (Table S3). In this study, these 26 genes serve as anchors in the co-expression networks to identify other candidate high-yield genes, which are not used for “re-identification”. After removing the redundancies, we retained 5746 genes, which we call feature genes, in matrix *X* (Figure 2).

The expression data (fragments per kilobase of transcript per million mapped reads (FPKM) values) of these feature genes were grouped into the matrix *X* (*X*_1_ for Taoyuan and *X*_2_ for Jinghong) (Figure 2B). In the first step of the DCT analysis, we factorized *X* into *W* and *H* using our proposed jcNMF, where one column of *W* represents a developmental gene (co-expression) module or pattern among rice tissue samples, and one row of *H* represents the tissue-specific gene set of each rice tissue. The computational algorithm for solving *H* and *W* as well as its convergence proof, is shown in the Supplementary material (Files S1 and S2). Different from conventional approaches, the advantage of jcNMF is able to directly represent the biological context, such as the conserved relationships or correlations among gene modules across tissues (*i.e.*, the conserved tissue correlations *W*_1_*W*_1_^T^ = *W*_2_*W*_2_^T^, rather than the conserved gene module compositions *W*_1_ = *W*_2_ in the case and control) (Figure 2C). This soft constraint on *W* well characterizes the biological and developmental relatedness in the rice samples, which were also supported by real data (see the conservation levels between tissues and genes in Figure 3). Specifically, using the 5746 feature genes, we assessed the conservation levels in the seven tissues and their gene sets between Taoyuan and Jinghong. In Figure 3, the correlations (*i.e.*, *WW*^T^) of the tissues between the Taoyuan and Jinghong samples (Figure 3B and C) were more consistently conserved than those found for the genes (Figure 3D and E). Therefore, in our DCT analysis, we set *W*_1_*W*_1_^T^ = *W*_2_*W*_2_^T^ = *R,* where *R* is the conserved correlation matrix for tissues obtained from Figure 3B and C, rather than simply *W*_1_ *= W*_2_ as in the traditional methods. Clearly, the hard constraint *W*_1_ *= W*_2_ is more restricted than the soft constraint *W*_1_*W*_1_^T^ = *W*_2_*W*_2_^T^ which is biologically meaningful based on the observed data (Figure 2).

**Figure 4:**
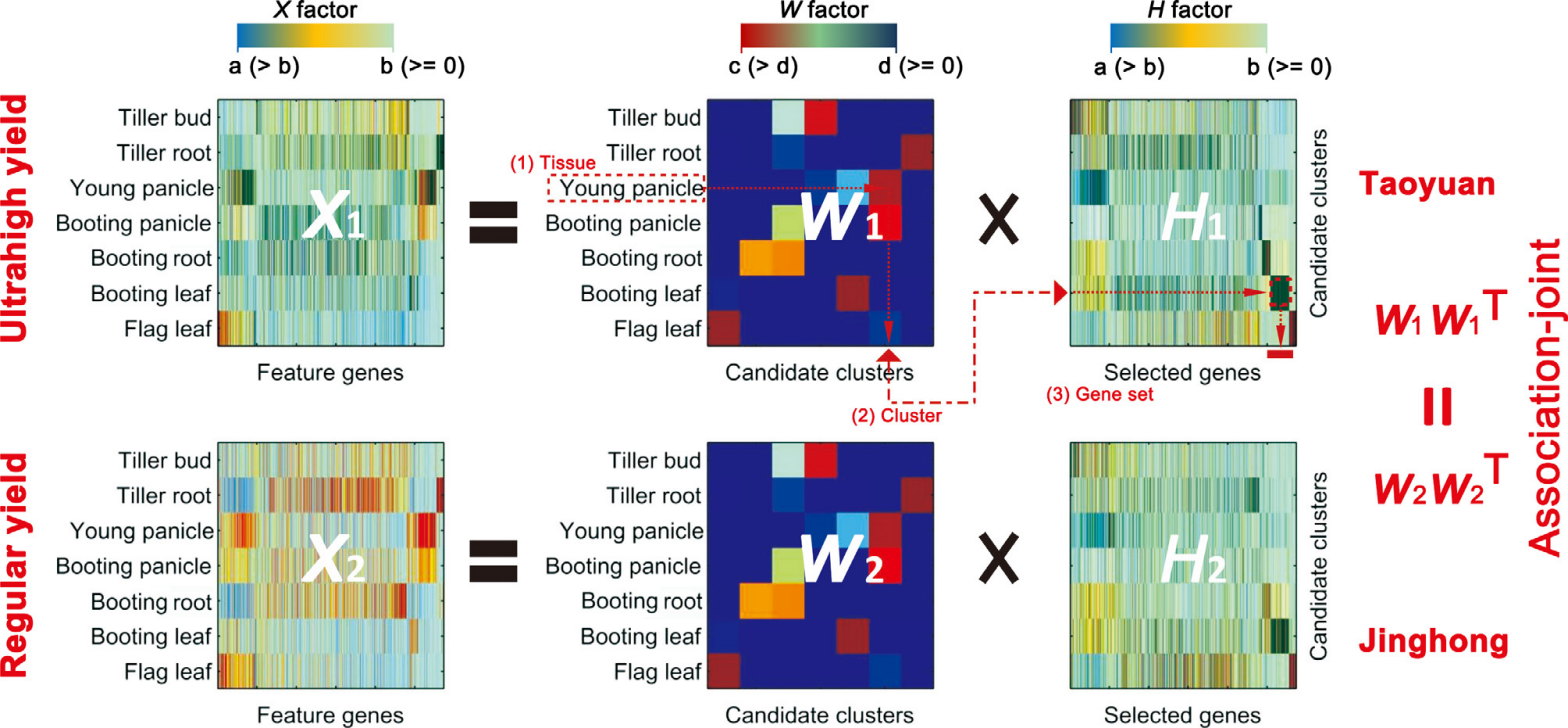
**Identification of gene sets for featuring a tissue** The diagrammatic sketch shows the process of identification, and the output from this step of the DCT analysis will be the input of the downstream construction of DEN for a tissue. Numerical output includes a group of triple array (tissue, cluster, and gene set), and biological output includes the genes characterized in each tissue. In this study, the rice variety 9311 seeds had the same genetic background, indicating unidentified environmental differences could leave their imprint in the epigenome and modify gene expression and regulatory networks. Thus, it is reasonable to assume that the tissue-related matrices *W*_1_ and *W*_2_ would also be consistent with the conserved correlations among tissues. *W*_1_, Taoyuan; *W*_2_, Jinghong and *H*_1_, Taoyuan; *H*_2_, Jinghong. In the color key, a, b, c, and d represent a non-negative number. [a, b] indicates the weight value range for gene, and [c, d] indicates the weight value range for tissue.

The second step of DCT is to construct the co-expression networks of genes. We calculated the Pearson’s correlation coefficient (PCC) between two columns/genes of either *H*_1_ or *H*_2_. Figure 4 provides a schematic of the approach used to obtain the gene set that will be used to construct co-expression networks for rice in one place. A tissue can be best characterized by a gene cluster. For example, the young panicle is best characterized by the sixth cluster in matrix *W*, which corresponds to the 5746 genes in the matrix *H*_1_ (Figure 4). We selected those genes with significantly higher weights than the mean of the sixth row, which formed a gene set accounting for the young panicle of Taoyuan. Furthermore, we calculated the correlation coefficients between each pair of genes, and those gene pairs with significant correlation coefficients formed co-expression networks. We conducted the same procedure for the Jinghong samples, and those gene pairs that were included in only one of the networks were used to construct DENs for a certain tissue (Figure 4). The DENs of all the tissues comprehensively accounted for the difference in the gene expression networks between Taoyuan and Jinghong rice throughout the growth process, and they link/bridge the internal gene expression patterns with the ultrahigh yield, *i.e.*, genotypes → DEN → phenotypes, in terms of the associations.

**Figure 5:**
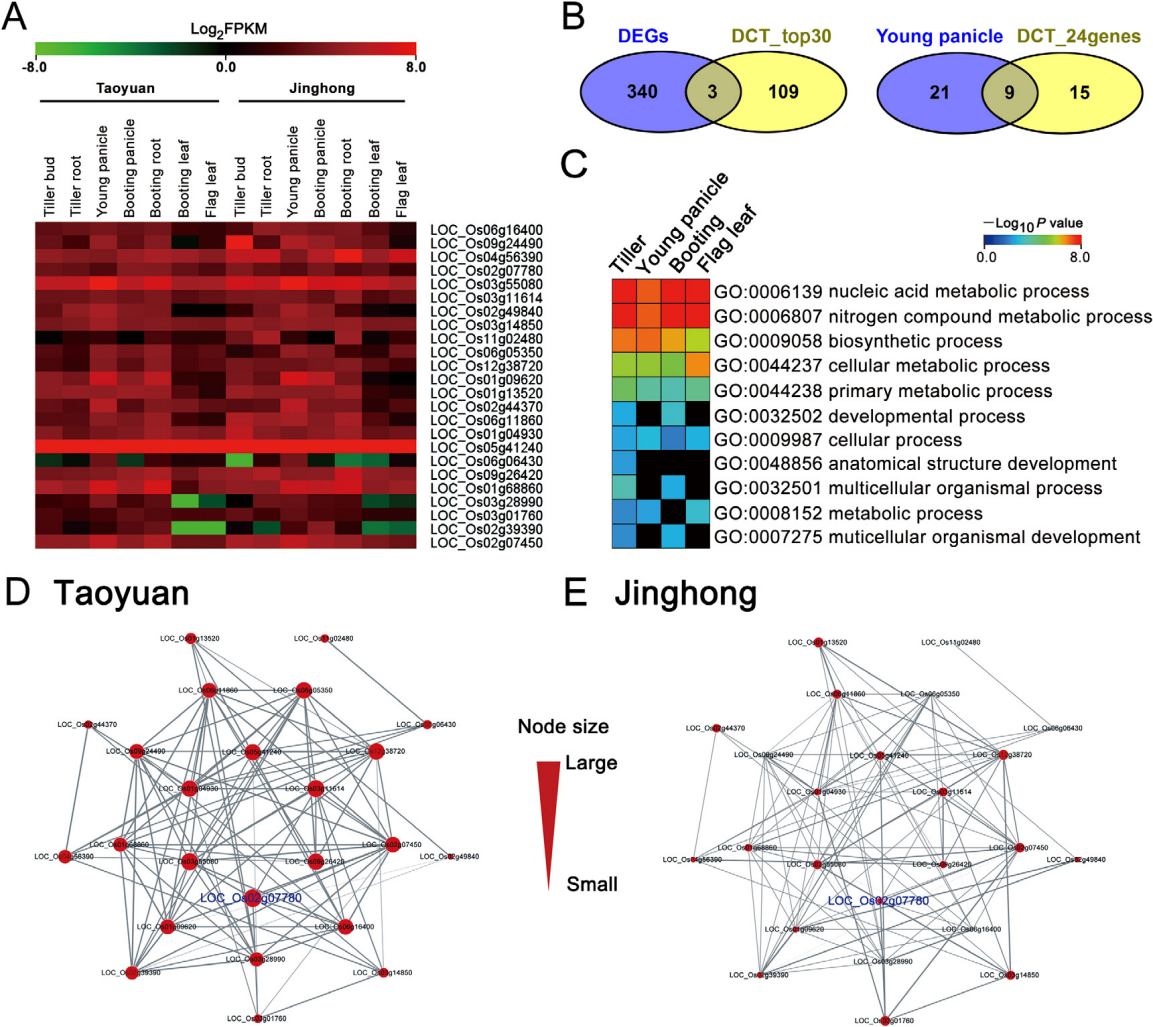
**Expression analysis and associative networks of the candidate high-yield associated genes screened by DCT analysis** **A.** Heatmap of the expression levels of 24 candidate high-yield genes selected from all the DENs of the tissues in the rice variety 9311. **B.** Venn diagram showing the overlap of DEGs and candidate genes screened by DCT analysis (total of the top 30 genes). The overlap of candidate genes in the young panicle (top 30 genes) and the final 24 candidate genes were screened by DCT analysis. **C.** GO enrichment of the top 30 genes was analyzed by DCT analysis at four developmental stages. The booting stage includes booting panicle, booting root, and booting leaf (Table S7). **D.** and **E.** DEN of the 24 top candidate genes associated with yield in Taoyuan rice (D) and Jinghong rice (E), respectively. Notably, more associations among genes were found in Taoyuan rice than those in Jinghong rice, indicating the genes in Taoyuan rice would have strong expression correlations. The thicker are the lines, the higher are the network degrees and the degrees of genes in Taoyuan rice are higher than those in Jinghong, indicating that one gene in Taoyuan rice would exhibit more interactions with partner genes (*i.e.*, more hub genes) than one gene in Jinghong rice on average.

Finally, the top candidates from the set of ultrahigh yield-associated genes (or key genes) were selected from the DEN of each tissue. The criterion used for this selection is the rank of the relatedness of a gene with prior-known yield-associated genes (*i.e.*, the *R*(*x*) value; see “Materials and methods”). To obtain a strong signal of high yield, in this study, we selected genes that were ranked in the top 30 based on the *R*(*x*) values (*i.e.*, based on the cross-tissue co-expressed network structure and state). We selected the top 30 candidate genes with highest differential associations in each tissue (Table S4). Then, 24 candidate high-yield genes among the top 30 genes were found in at least four of the seven tissues, but their expression levels were not significantly difference between Taoyuan and Jinghong rice based on the traditional differential expression method (Figure 5A, Tables S2 and S5). In total, 112 candidate genes were screened by DCT analysis, and only three DEGs overlapped (Figure 5B). Particularly, nine of 24 candidate high-yield genes were identified in the young panicle using DCT analysis (Figure 5B).

**Supplementary Figure S2:**
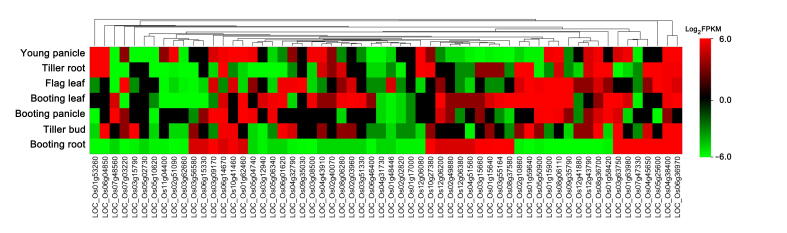
**Transcription factors in the key module analyzed by dynamic cross-tissue analysis and their expression in seven tissues**.

The 24 high-yield candidate genes identified using the DCT algorithm showed large association (network) changes but moderate expression changes with fold changes only larger than 1.2. This explains why they would be disregarded by the traditional differential expression analysis method that considers only significant expression changes (mainly fold change >2). Additionally, the key TFs were screened by the DCT analysis to reveal their roles in the regulation network of candidate high-yield genes (Figure S2 and Table S6). Gene ontology (GO) enrichment analysis also showed that these 24 candidate genes were involved in “nitrogen compound metabolic process” (Figure 5C and Table S7). The DEN of the 24 candidate genes showed that they exhibited more associations with the yield-associated genes at Taoyuan than that at Jinghong (Figure 5D and E, Figure S3). These results further supported that most of these 24 genes might play important roles in the ultrahigh yield of Taoyuan rice, and are probably the key network-hubs controlling the yield.

### Comparison of jcNMF with other models

**Figure 6:**
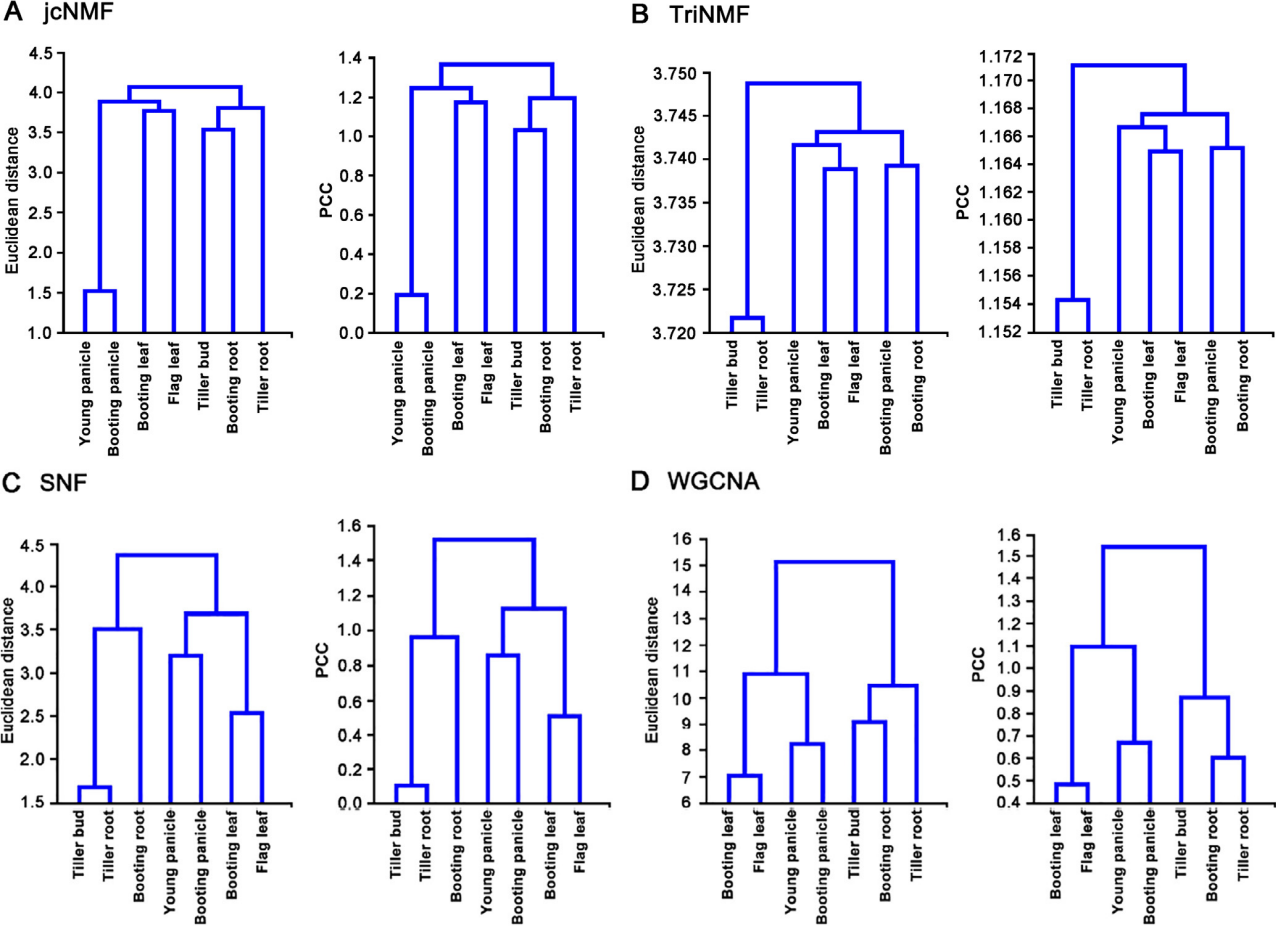
**Comparison of the conservation of tissue associations (Euclidean distance or Pearson correlation) using different methodsA.** jcNMF. **B.** TriNMF. **C.** SNF. **D.** WGCNA. Comparisons with additional methods are shown in the Figure S4. jcNMF, joint-correlation non-negative matrix factorization; TriNMF, conventional NMF-based method; SNF, similarity network fusion; WGCNA, weighted correlation network analysis; PCC, Pearson’s correlation coefficient.

On the one hand, as the core of the DCT algorithm, jcNMF has a similar ability to that of conventional NMF to capture the local pattern during dimension reduction. Although the analyzed expression data contain seven tissues and *X* is actually a low-rank matrix, the local pattern (*i.e.*, tissue conservation) rather than dimension reduction (*i.e.*, gene filtering) would be the main target using jcNMF. On the other hand, one main merit of jcNMF is to reflect the conserved tissue associations during integrative data analysis based on the proposed soft constraint. To evaluate the efficiency of jcNMF, several typical integration methods have also been applied and compared according to their influence on the tissue associations caused by corresponding data transformations. Simply, the tissue or sample association can be directly shown and compared as hierarchical trees, as shown in Figure 6.

**Supplementary Figure S4:**
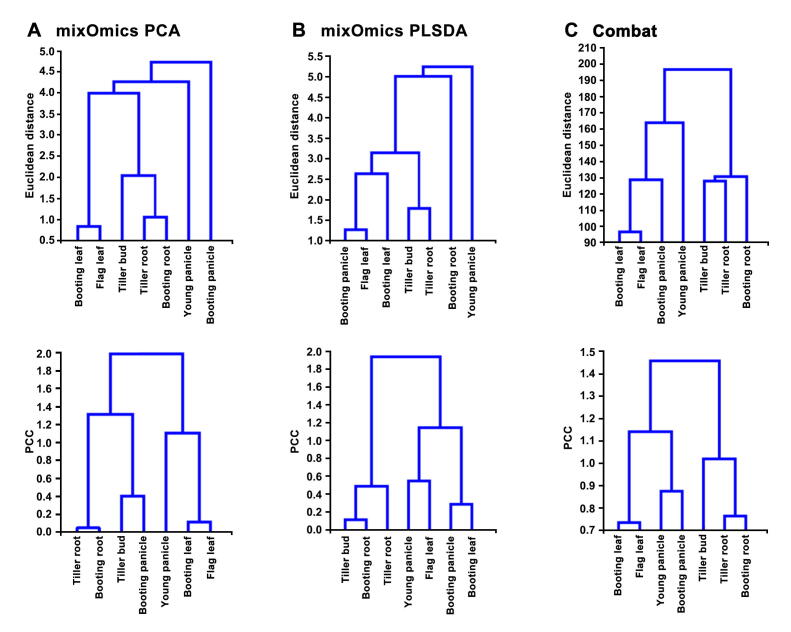
**Comparison of the seven tissue associations (Euclidean distance or Pearson’s correlation) using three different methods.** mixOmics PCA **(A)**, PLSDA **(B)**, Combat **(C)**. PCC, Pearson’s correlation coefficient; mixOmics PCA, mixOmics principal component analysis; PLSDA, partial least squares-discriminant analysis.

Obviously, jcNMF can reflect or recover the tissue associations based on the Euclidean distance or Pearson’s correlation, *e.g.*, two panicle samples would be clustered together; and two leaf samples would be also clustered together (Figure 6A). By contrast, all other methods have certain limitations: (i) the conventional NMF-based method (TriNMF) ignores the associations between root and panicle samples after matrix factorization, although leaf samples can be clustered together (Figure 6B); (ii) mixOmics principal component analysis (mixOmics PCA) was applied for feature reduction, but the association between panicle samples were also missed (Figure S4A); (iii) partial least squares-discriminant analysis (PLSDA) is a supervised method but confuses the tissue associations due to data transformation (Figure S4B); (iv) the batch-effect removing approach Combat would change the association between the root or panicle samples after adjusting data variances (Figure S4C); and (v) the pattern fusion method similarity network fusion (SNF) clusters leaf samples well, but it still shows some confusing associations between the root and panicle samples (Figure 6C).

The well-known weighted correlation network analysis (WGCNA) was also used in this study, although the number of samples in this work was actually less than that generally required by WGCNA. The WGCNA results were similar to those obtained with jcNMF, but the former approach still sensitive to the clustering distances used in the analysis (*i.e.*, the subtree among the bud and root samples showed only a slight change when different cluster distances were used) (Figure 6D). And its detected modules cannot be associated with the case-control samples according to the trait-association test. Thus, it will be difficult to perform follow-up gene selection and function analysis under this condition.

Therefore, jcNMF outperforms other existing integrative data analysis methods to maintain the biological context (*i.e.*, tissue conservation) in the integrative data analysis, and thus, the DCT algorithm is better able to discover downstream genes, modules, networks, and functions.

### Young panicles play an important role in the ultrahigh yield of Taoyuan rice

**Figure 7:**
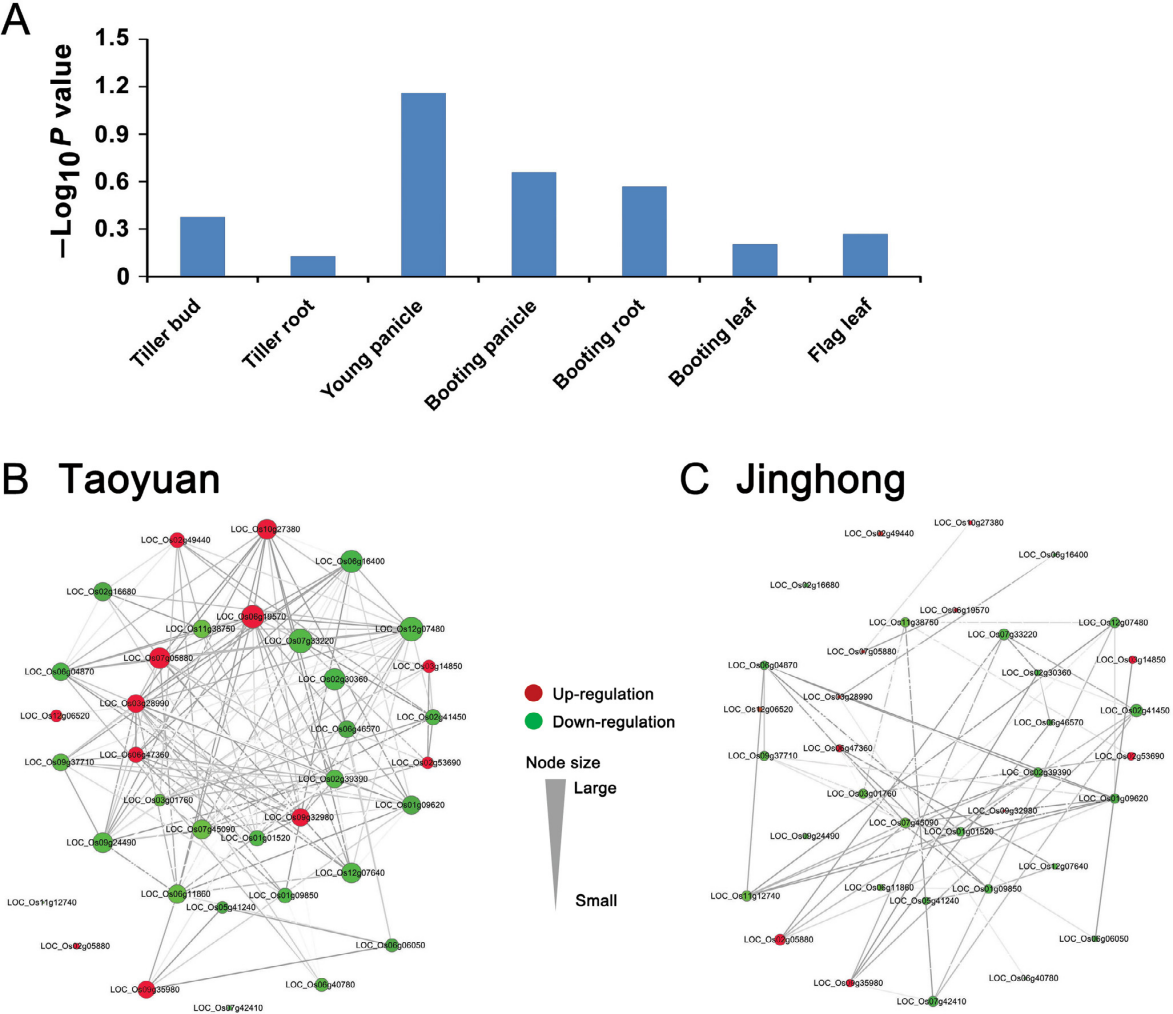
**The young panicle plays an important role in the ultrahigh yield at Taoyuan** **A.** Degree of gene co-expression network changes for each tissue between Taoyuan and Jinghong rice. *P* values were obtained using the matched pair *t*-test. **B.** and **C.** DEN between the young panicle highly ranked top 30 genes and six reported yield-associated genes (LOC_Os11g12740, LOC_Os02g05880, LOC_Os09g35980, LOC_Os07g42410, LOC_Os06g40780, and LOC_Os06g06050) (See Table S3) in the young panicle. Structure of the co-expression network in Taoyuan rice (B). Structure of the co-expression network in Jinghong rice (C). In the networks, the red nodes represent the up-regulated genes, and the green nodes represent the down-regulated genes. Notably, more associations were identified between genes in Taoyuan rice than between those in Jinghong rice, indicating that the genes in Taoyuan rice would exhibit strong expression correlations. Additionally, the network degrees of genes in Taoyuan rice were high compared with those in Jinghong rice, indicating that one gene in Taoyuan rice would have more interactions with partner genes (more hub genes) than that in Jinghong.

We further evaluated the node degree of DENs in each tissue to identify the tissue whose DEN showed the most significant change between Taoyuan and Jinghong rice as determined using the matched pair *t*-test. Interestingly, the young panicle exhibited the lowest *P* value (Figure 7A), indicating that it is the major tissue that causes the greatest changes in the gene co-expression networks for the ultrahigh yield of rice at Taoyuan. For the young panicle, the DEN of the top 30 candidate genes associated with yield-associated genes at Taoyuan and Jinghong was reconstructed (Figure 7B and C). Clearly, there are high associations between our selected candidate genes and prior-known yield-associated genes (Table S3) in the module-based co-expression network. In Taoyuan specific networks, there are many module genes associated with known yield-associated genes LOC_Os09g35980 (*TAC1*), LOC_Os06g40780 (*MOC1*) or LOC_Os06g06050 (*D3*) (*e.g.*, nodes with large degrees in network visualization), for example, the candidate genes LOC_Os05g41240 (MYB), LOC_Os06g11860 (*ERF*), and LOC_Os03g28990 (zinc finger) (Figure 7B). Furthermore, the prior-known yield-associated genes LOC_Os09g35980 and LOC_Os06g06050 also exhibited significant associations in Taoyuan rice (Figure 7B). The increased number of associations among candidate genes and yield-associated genes in Taoyuan rice compared with those in Jinghong rice can be considered to have a stronger driving influence on ultrahigh yield (Figure 7B and C).

The importance of the young panicle can be further supported by the results of the GO enrichment analysis of the top 30 candidate genes from the young panicles (Figures 5C, S5, and Table S7). Compared with other tissues, the composition of GO enrichments from the young panicle is very similar to that from the previously reported 26 yield-associated genes (Figure S5). Many of the candidate genes in the young panicle are involved in “nitrogen compound metabolic process” (*P* = 0.000084) (Figure S5 and Table S7). Moreover, nine of the final 24 candidate high-yield associated genes screened from the seven tissues by the DCT analysis were found in the young panicle, and this number was higher than those found in the other tissues (Figure 5B, Tables S4 and S5). Thus, these key genes in the young panicle would not only exhibit expression associations with the known yield-associated genes, but also functional similarity with yield-associated genes.

### Functional validation of candidate genes

**Supplementary Figure S6:**
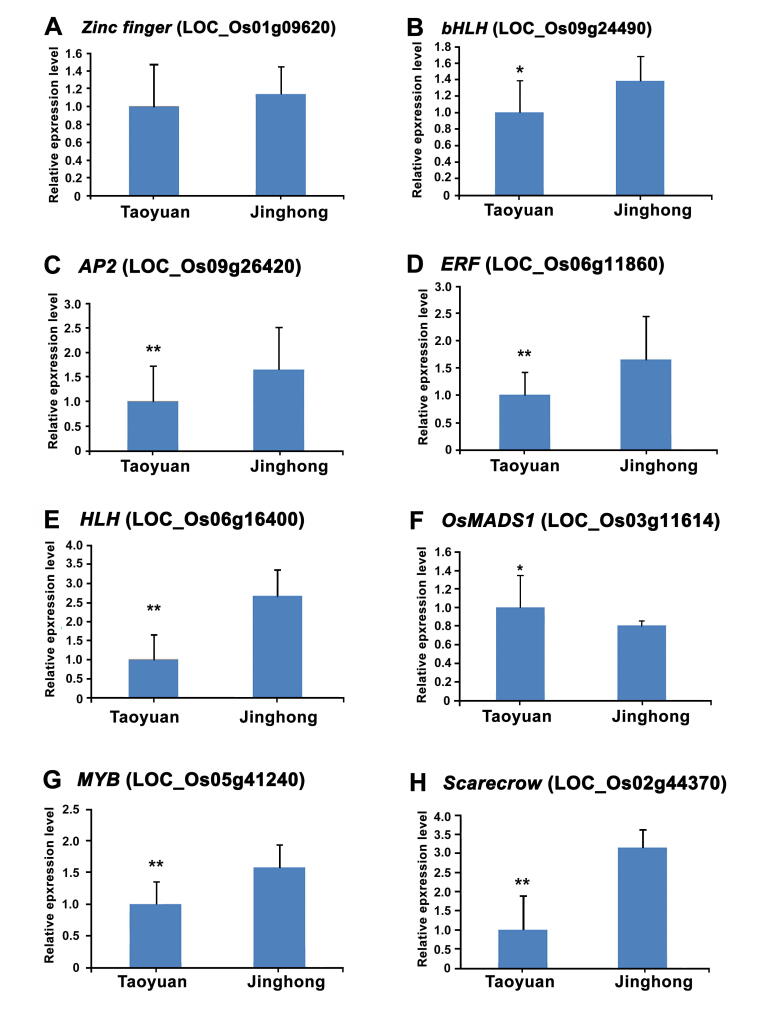
**Quantitative RT-PCR validation of eight candidate high-yield-associated genes in the young panicles of rice variety 9311.** The differentially expression patterns of zine finger (LOC_Os01g09620) **(A)**, bHLH (LOC_Os09g24490) **(B)**, AP2 (LOC_Os09g26420) **(C)**, ERF (LOC_Os06g11860) **(D)**, HLH (LOC_Os06g16400) **(E)**, OsMADS1 (LOC_Os03g11614) **(F)**, MYB (LOC_Os05g41240) **(G)** and scarecrow (LOC_Os02g44370) **(H)**. The asterisks indicate significant differences in the gene expression levels between Taoyuan and Jinghong as determined by Student’s *t*-test (*, *P* < 0.05; **, *P* < 0.01).

We firstly used qRT-PCR to validate the changes in the expression levels of eight candidate high-yield associated genes in the young panicle samples of the rice variety 9311, including *OsMADS1* (LOC_Os03g11614) and AP2 transcription factor (LOC_Os09g26420) (Figure S6). Interestingly, three MADS box genes, *OsMADS1* (LOC_Os03g11614), *OsMADS57* (LOC_Os02g49840), and *OsMADS72* (LOC_Os03g14850), were identified as candidate high-yield associated genes by the DCT analysis in our study. Previous studies have reported that MADS-box genes encode TFs that are involved in reproductive development, including flowering induction and flower meristems as well as in the regulation of fruit, seed and embryo development [15], [16], [17]. Our qRT-PCR results consistently showed that the expression of *OsMADS1* was up-regulated in Taoyuan rice (Figure S6 and Table S5). AP2 have been reported to be involved in rice starch biosynthesis and the improvement of grain yield under stress [18], [19]. In our study, *AP2* (LOC_Os09g26420) was also identified to be a candidate high-yield associated gene by DCT analysis, and its expression level in young panicles was validated by qRT-PCR (Figure S6). These results showed that our transcriptome data are reliable.

**Figure 8:**
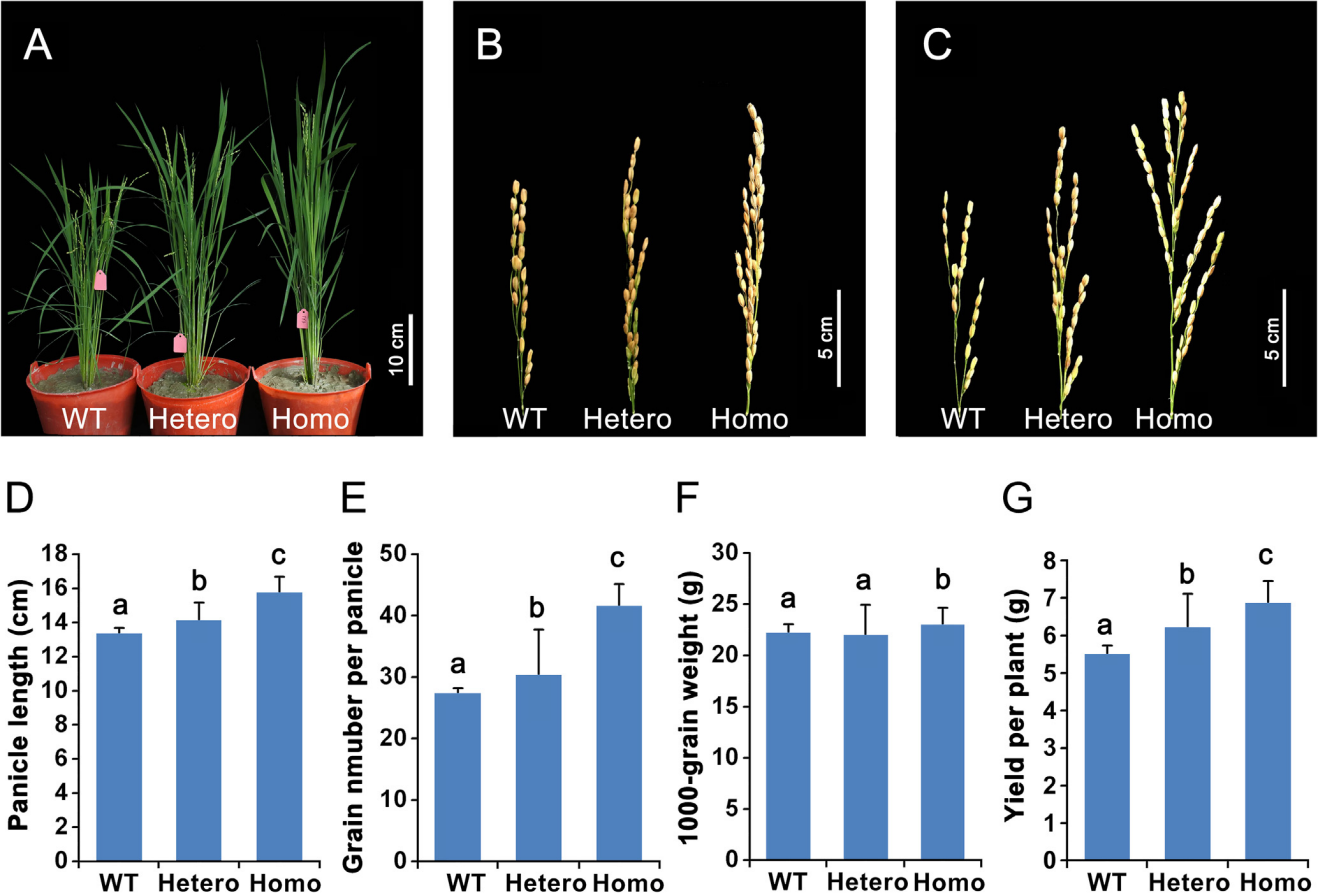
**CRISPR**/**Cas9 experimental validation of a previously functionally unknown gene (*OsSPL4*) identified as a key high-yieldassociated gene by our DCT analysis** The 3-bp deletion mutation heterozygous and homozygous plants were produced by CRISPR/Cas9, and these plants are shown as Hetero and Homo in the figures, respectively. The following phenotypes were compared between the *OsSPL4*-edited (Hetero and Homo) and WT plants. **A.** Plant architecture, scale bar = 10 cm. **B.** Panicle phenotype, scale bar = 5 cm. **C.** Panicle phenotype showing the grain number, scale bar = 5 cm. **D.**–**G.** Different agronomic trait results between the *OsSPL4*-edited (Hetero and Homo) and WT plants: panicle length (cm) (D), grain number per panicle (E), 1000-grain weight (g) (F), and yield per plant (g) (G). Significant difference between WT, Hetero, and Homo plants was determined using ANOVA. Groups carrying the same letter of a, b, or c show no significant difference while significant difference is found between groups carrying the different letter of a, b, or c (*P* < 0.05).

**Supplementary Figure S7:**
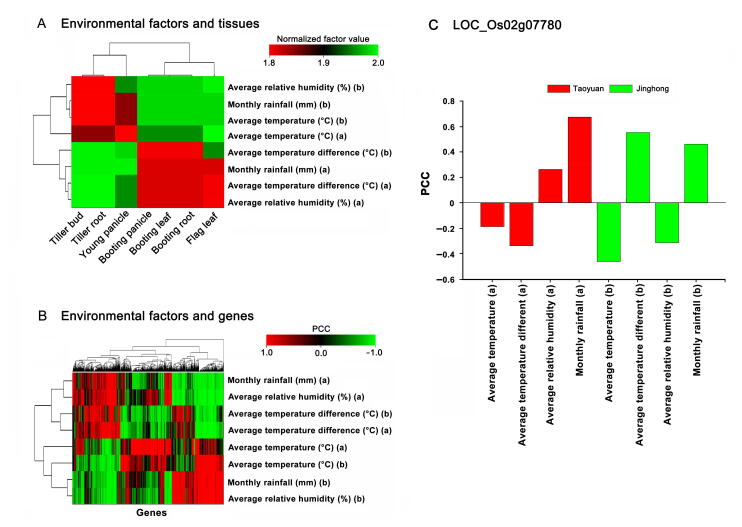
**Environmental factors and gene expression. A.** Clustering of environmental factors based on their observed values, *e.g.*, in the heatmap matrix, each element represents the value of one environmental factor tested at one sampling time. Here, (a) represents the environmental factor observed at Taoyuan, and (b) represents the environmental factor observed at Jinghong. Other factors have similar labels. **B.** Clustering of environmental factors based on their associations with genes, *e.g.*, in this heatmap matrix, each element indicates PCC between one environmental factor and one gene in the samples from Taoyuan (a) or Jinghong (b). **C.** The associations between environmental factors and a candidate key yield-associated gene LOC_Os02g07780 (*OsSPL4*). This gene is negatively associated with the average temperature difference but positively correlated with average relative humidity at Taoyuan, which is reversed at Jinghong. Thus, these factors would reflect important environmental differences between Taoyuan (a) and Jinghong (b), and the associated gene *OsSPL4* is likely a candidate key yield-associated gene responding to such environmental changes. PCC, Pearson’s correlation coefficient.

To solidly validate the candidate key genes identified by DCT analysis, we further edited the *OsSPL4* (LOC_Os02g07780) gene via CRISPR/Cas9. Sequencing analysis of the targeted site revealed a 3-bp heterozygous deletion mutation produced by CRISPR/Cas9 in the T0 plants (Figure 8). We further obtained heterozygous, mutation-homozygous and wild type (WT) plants in the T2 segregation population. We compared the phenotypes between the *OsSPL4*-edited (both heterozygous and homozygous) T2 lines and WT plants (Figure 8). The plant heights of the *OsSPL4*-edited lines were slightly increased (Figure 8A), and the Cas9-edited plants exhibited longer panicles and a larger number of grains per panicle than WT plants (Figure 8B–G). Strikingly, for our primary analysis, the yield of these homozygously mutated plants was significantly higher than that of the WT plants (Figure 8G). The expression level of *OsSPL4* was down-regulated in Taoyuan rice (Table S5) and the rice variety 9311 at Taoyuan also exhibited a higher grain number per panicle than that at Jinghong, implying that *OsSPL4* is a key gene for the ultrahigh yield at Taoyuan (Figure 8 and Table 1). In addition, the association analysis between environmental factors and LOC_Os02g07780 (*OsSPL4*) showed that the expression level of this gene is negatively associated with the average temperature difference but positively correlated with the average relative humidity at Taoyuan (Figure S7C), indicating that this gene is indeed a regulatory factor responding to environments. *OsSPL4* is an SBP-box gene and previous studies have shown that some SBP-box genes were involved in panicle development and yield in rice [20], [21], [22], [23]. This study provides the first demonstration that *OsSPL4* is a key regulatory gene in the ultrahigh yield of Taoyuan rice, and shows that our DCT is an effective method to identify key genes and networks affecting the formation of a complex trait.

## Discussion

To our knowledge, this is the first systematic analysis of the multiple tissues of rice across developmental stages, and we attempted to integrate the transcriptomic data with the aim to identify key genes and networks for agronomic traits in plants. The environment affecting the same genome with different characteristics has been widely documented in many organisms, such as twins [24], yet the mechanisms in plants have not been well elucidated. As an interesting case, we observed the ultrahigh yield of rice at Taoyuan, which was found to be at least 70% higher rice yield than that in the control area under the same cultivation management over the 4 years of field experimentation.

To reveal the internal key genes and their modules underlying the ultrahigh yield at the network level, we developed a dynamic meta-analysis framework across tissues and developmental stages, *i.e.*, the DCT algorithm with jcNMF, which can construct the associations of tissues and gene modules to a specific phenotype (ultrahigh yield in this study) by integrating gene expression profiles within the biological context. Notably, we identified the gene-modules by conducting the study on the cross-tissue and multi-developmental stages. Based on our model, the gene compositions of those gene-modules were conserved across tissues, but their expression levels were generally different depending on the tissues. Indeed, as a matrix decomposition based approach, jcNMF can not only capture the local pattern from expression data (*i*.*e*., capturing gene modules) in a standard manner, but also maintain the global pattern in a biological context (*i*.*e*., reserving tissue conservations) in a new way, which is implemented as Figure 4 and supported by the comparisons shown in Figure 6. In addition, the follow-up differential network analysis of gene modules can reveal molecular details of key genes at the network level rather than at the expression level, which would be more efficient than the conventional WGCNA method (Figure 5, Figure 6). Overall, the DCT algorithm is a powerful computational method for cross-tissue biological data analysis. It could be extended to other general integrative analysis by considering various types of fundamental matrix decomposition models and categories of temporal-spatial contexts/constraints.

Supporting the DCT analysis here, the panicle size and branch number in a panicle are directly associated with the rice productivity. A previous study reported that *OsSPL14* which is highly expressed in the young panicle can increase the primary branches of panicle, leading to a high yield in rice [20], [21]. In the present study, the candidate yield associated gene *OsSPL4*, which is another *SPL* gene, confirmed that this gene also increases the grain number of panicle and grain yield (Figure 8). The *OsSPL4* should be the key gene for ultrahigh yield observed in Taoyuan rice due to its contribution of the large number of panicles and grain number per panicle (Figure 8 and Table 1). In addition, Taoyuan has different climates with large temperature differences after heading and slightly low humidity throughout the growing period (Table S1). In the present study, *OsSPL4* was identified with significant associations with these environmental factors (Figure S7).

In our systematic study, both of our selected 24 candidate genes and 26 prior-known yield-associated genes were enriched in two significant pathways with the GO terms as “nitrogen compound metabolic process” and “nucleic acid metabolic process”. Particularly, many of the candidate genes discovered in the young panicle are involved in “nitrogen compound metabolic process” (*P* = 0.000084) (Figure S5 and Table S7), implying that these candidate genes are involved in nitrogen metabolism. It has been documented that nitrogen is actually a major driving force for crop yield improvement, and nitrogen absorption and metabolism can affect rice growth and production [25]. In our study, one candidate gene LOC_Os11g02480, which encodes *WRKY46,* was identified as the key gene (Table S5). It has been reported that *OsWRKY46* is involved in the iron stress response and the promotion of leaf development, whereas excess Fe may cause yield loss due to leaf bronzing in rice [26]. Another study has reported that the expression level of *WRKY46* was induced in the rice leaf sheath under N-starvation [27]. Therefore, the 24 candidate genes identified in our study should be the key genes for the ultrahigh yield of Taoyuan rice. Further studies on these genes may provide more genetic resources for a high yield of rice.

Lately, *OsMADS1* has been identified to be a key gene of the rice grain yield quantitative trait locus *qLGY3*, which is a key downstream effector of G-protein. The alternatively spliced protein *OsMADS1^lgy3^* was confirmed to be associated with the formation of long grains, which results in an increase in the grain yield of rice [28]. In rice, the overexpression of *OsMADS57* can increase tillers, and the expression level of *OsMADS57* in Taoyuan rice was higher than that of Jinghong rice at the tillering stage, suggesting that *OsMADS57* is one of the key yield-associated genes for Taoyuan rice (Table S5) [17]. As one of the targets of miR444, the expression level of *OsMADS57* was decreased under N- or P-starvation, which indicates that *OsMADS57* plays a role in rice nitrate-signaling pathway [29].

In summary, this study developed a systems biology approach to identify both the key tissue and high-yield associated genes, and to elucidate the associations between the gene expression network and the ultrahigh yield of rice at Taoyuan. The results shed novel light on our understanding of the genetics of the ultrahigh yield of rice, or even all Gramineae crops. The DENs and key candidate high-yield genes provided rich information to achieve a much higher yield in rice and other Gramineae crops by artificially regulating or perturbing the identified gene networks. In this work, we mainly considered network information for the identification of high-yield genes, and as one future topic, we can further explore dynamic information, such as dynamic network biomarker (DNB) [30], [31], [32], [33], [34], [35], from time-course data to improve the approach in terms of effectiveness and efficiency. Importantly, the DCT analysis approach could also be applied to other plant or animal systems if different phenotypes under various environments with the common genome sequences of the examined sample, such as twins or plants exposed to stress conditions.

## Materials and methods

### Plant materials and field experiments

Field experiments using the rice variety 9311 were conducted in 2010, 2011, 2013, and 2014 at Taoyuan and Jinghong, Yunnan in China (Figure S1F and Table 1). We chose Jinghong, which is a typical *indica* rice plating region, as the control place. The same crop management practice was strictly used, such as the same plot area, planting density and fertilized nitrogen use (225 kg·N·ha^−1^) at the two places. We set up 3–4 replicates of 15 m^2^ plots and conducted careful phenotyping throughout the growth period. Water, weeds, insects, and disease were controlled because their control is needed to avoid yield loss.

### RNA-sequencing and data processing

Tiller buds, tiller roots, young panicles, booting panicles, booting leaves, booting roots, and flag leaves of the rice variety 9311 from Taoyuan and Jinghong were collected, immediately frozen in liquid nitrogen and then kept at −80 °C. Total RNA of the tissues was extracted and determined using the NanoDrop ND-2000 system (Thermo Scientific, Waltham, MA), followed by sequencing using an Illumina HiSeq 2500 platform. Raw reads were filtered by in-house Perl script, and then clean reads were used for further analysis. The clean reads were performed using the TopHat and Cufflinks package [36], [37]. The transcript levels were qualified as FPKM generated by Cufflinks [36]. Then, bioinformatics analysis in this study was conducted, and the work routine is shown in the flow chart as Figure 1.

### DCT network analysis

To integratively analyze the factors affecting the high yield of rice at Taoyuan, we developed a computational algorithm of the DCT network analysis to study multiple tissues and multi-developmental stages of rice as described below (File S1 and File S2).

Using RNA sequencing techniques, transcriptome data analysis produced the numeric matrix of FPKM values of rice genes, where *X* denotes the dataset of Taoyuan or Jinghong, ${\text{x}}_{\text{mn}}$ denotes the FPKM value, *n* is the number of genes, and *m* is the number of sampled tissues:

(1) $$X=\left(\begin{array}{ccc} {\text{x}}_{11} & \cdots & {\text{x}}_{1\text{n}}\\ \vdots & \ddots & \vdots \\ {\text{x}}_{\text{m}1} & \cdots & {\text{x}}_{\text{mn}} \end{array}\right)$$

The first step of DCT is matrix factorization. Because the FPKM and many other types of biological data are non-negative, NMF is widely used [38], [39] to analyze such data, and *W* and *H* are two factor matrices of *X*:

X=W·H,

where the solution is in the following format:

(2) $$W=\left(\begin{array}{ccc} {\text{w}}_{11} & \cdots & {\text{w}}_{\text{1k}}\\ \vdots & \ddots & \vdots \\ {\text{w}}_{\text{m}1} & \cdots & {\text{w}}_{\text{mk}} \end{array}\right)\;\;\;\;\;\;\;\;\;\;\;\;\;H=\left(\begin{array}{ccc} {\text{h}}_{11} & \cdots & {\text{h}}_{1\text{n}}\\ \vdots & \ddots & \vdots \\ {\text{h}}_{\text{k}1} & \cdots & {\text{h}}_{\text{kn}} \end{array}\right)$$

Here, the biological meaning of *W* is the gene-modules (or networks) of samples (or individuals) (*i.e.*, the developmental gene co-expression patterns among rice tissues), and the biological meaning of *H* represents the gene-module expressions among samples corresponding to phenotypes (*i.e.*, the tissue-specific gene-module levels of each rice tissue). Note that *X* is the observed data, whereas *W* and *H* are unknown variables to be solved.

Many algorithms based on NMF were developed to solve *W* and *H*, typically, joint non-negative matrix factorization (jNMF) [9], [10], [40], which prescribes the same W of two or more NMF equations as a hard-constraint:

(3) $$\left\{\begin{array}{c} X_{1}=W_{1}\cdot H_{1}\\ X_{2}=W_{2}\cdot H_{2}\\ W_{1}=W_{2} \end{array}\right)$$

where note that $W_{\text{1}}\;\text{=}\;W_{\text{2}}$, implying the conserved tissue compositions in terms of gene-modules in case samples *W*_1_ and control samples *W*_2_.

In contrast, in DCT, we used a new jcNMF to factorize *X* into *W* and *H*:

(4) $$\left\{\begin{array}{c} X_{1}=W_{1}\cdot H_{1}\\ X_{2}=W_{2}\cdot H_{2}\\ W_{1}W_{1}^{T}=W_{2}W_{2}^{T} \end{array}\right)$$

The advantage of jcNMF is to make the relationships between each lines (tissues) in *W*_1_ be the same as those in *W*_2_, so that tissues more closely related are presented by lines, more similar to each other in each *W*. The biological meaning of this soft-constraint is that *W* should be consistent with biological and developmental relatedness. Clearly, the hard constraint *W*_1_ = *W*_2_ is stronger (or more restricted) than the soft constraint *W*_1_*W*_1_^T^ = *W*_2_*W*_2_^T^ which is biologically meaningful based on the observed data (Figure 3). In this way, jcNMF indicates that the conservation of expression correlation between tissues should be carefully considered rather than the conservation of the expression levels of tissues during the cross-tissue integrative analysis. The jcNMF algorithm resolving this equation and its convergence proof are shown in the File S1 and S2, respectively.

Then, the second step of DCT is to construct the co-expression networks of genes. We calculated the PCC between each two columns/genes of either *H*_1_ or *H*_2_:

(5) $$\text{PCC}\left(\text{A},\text{B}\right)=\frac{1}{k-1}\sum_{i=1}^{k}\left(\frac{a_{i}-\overset{¯}{a}}{{\overset{}{\sigma }}_{a}}.\frac{b_{i}-\overset{¯}{b}}{{\overset{}{\sigma }}_{b}}\right)$$

where $a_{i}$ belongs to column A, and $b_{i}$ belongs to column B of *H*, respectively. $\sigma_{a}$ and $\sigma_{b}$ are the standard deviations of column A and column B, respectively. If two columns/genes are significantly correlated (*P* < 0.05), they are placed in the gene co-expression network. Eventually we constructed the gene co-expression networks of *H*_1_ and *H*_2_ for the case and control samples, named *C*(*H*_1_) and *C*(*H*_2_), respectively. The difference sets *C*(*H*_1_) − *C*(*H*_2_) and *C*(*H*_2_) − *C*(*H*_1_), named *Diff*_1_ and *Diff*_2_, respectively, represent the case/control specific gene co-expression network, *i.e.*, the DEN [11], [12], [41].

The final step involves the selection of the potentially phenotype-associated genes (or so-called key genes). The main criterion used in this selection is the rank of the relatedness of a gene with prior-known to be phenotype-associated genes (here the phenotype is just the yield of rice):

(6) $$R\left(u\right)=\frac{\Sigma_{v\in S}\text{PCC}\left(u,v\right)}{\left|S\right|}$$

*S* is the set of the known yield associated genes, *v* is a gene in set *S*, and *u* is the gene of interest. We selected genes whose *R*(*x*) value in the top 30 as the final results of the DCT analysis based on integrative consideration of each sample/tissue, and they can be further ranked by the product rank of *R*(*x*), expression fold-change and co-expression network degree.

In addition, to evaluate the structural changes in the tissue-specific co-expression network between Taoyuan and Jinghong, the *P* values of the degree changes of the genes in co-expression network are calculated. For each tissue, given a kind of feature genes (*e.g.*, TFs, yield-associated genes or DEGs), their degrees in the co-expression network from Taoyuan (*i.e.*, a degree vector D_Taoyuan_) should be different from those in the co-expression network from Jinghong (*i.e.*, a degree vector D_Jinghong_). This difference is evaluated by the *P* value of the matched-pair *t*-test on two numeric vectors D_Taoyuan_ and D_Jinghong_. The matched-pair *t*-test to evaluate the degree of differential DENs in each tissue was performed using MATLAB 2012a [42]. And the formula for a paired *t*-test:

(7) $$t=\frac{\sum d}{\sqrt{\frac{n\left(\sum d^{2}\right)-{\left(\sum d\right)}^{2}}{n-1}}}$$

where *d* = sum of the differences in the vector elements. For the young panicle, such degree changes are significantly observed in all three types of feature genes. Particularly, compared with other tissues, significant network-degree changes of the reported yield genes and yield-associated genes in DEN are only observed in the young panicle. Thus, it would be a high priority to further investigate the associations among transcription factors, yield-associated genes and DEGs to identify the candidate key genes driving the ultrahigh yield observed in Taoyuan rice.

### Functional enrichment analysis

Functional annotations of the DEGs and candidate genes were performed to search against the GO database [43]. The top 30 candidate high-yield genes of each tissue from DCT network analysis were also analyzed by GO enrichment analysis. The results of GO annotations were submitted to AgriGO for enrichment analysis, and GO terms with corrected FDR < 0.05 were considered to be significantly enriched [44].

### qRT-PCR validation

The expression levels of eight high-yield candidate genes were randomly selected to be verified by qRT-PCR using the same RNAs that was used for RNA-seq [45]. Rice gene *actin1* was used as the internal control for qRT-PCR analysis (Table S8). And then real-time RT-PCR was performed on an ABI StepOne Real-Time PCR System (Applied Biosystems, Foster City, CA) with three replicates using a FastStart Universal SYBR Green Master (Roche, Mannheim, Germany). The relative expression level was normalized and quantified using the 2^−△△^CT method [46]. Significant differences of the expression levels between Taoyuan and Jinghong samples were evaluated using Student's *t*-test (*, *P* < 0.05; **, *P* < 0.01).

### CRISPR/Cas9 editing of ***OsSPL4***

To edit *OsSPL4*, a 20-bp sequence (5′-AGGTGAGGTGCCAGGTGGAA-3′) in the exon of the gene was selected as the target of the guide RNA (gRNA) using the CRISPR-P tool (http://rice.hzau.edu.cn/cgi-bin/rice/CRISPR_rice) [47]. Synthetic oligonucleotides containing the target and adaptor sequences were annealed and then subcloned into the *Aar*I restriction sites of the gRNA cloning vector (Table S8). The construct was introduced into the *Agrobacterium tumefaciens* strain EHA105 by electroporation, and positive agrobacteria were used to infect rice Nipponbare callus as previously described [48]. After the regeneration of plants, the target region was sequenced to screen for mutants, and T2 homozygous, heterozygous and wild type of *OsSPL4* lines were identified for phenotyping.

## Data availability

The sequencing data for this project have been deposited in the Genome Sequence Archive [49] at the National Genomics Data Center, Beijing Institute of Genomics, Chinese Academy of Sciences / China National Center for Bioinformation (GSA: (CRA002804), and are publicly accessible at http://bigd.big.ac.cn/gsa. The data are also at the NCBI Sequence Read Archive (SRA: SRP213003).

## Code availability

DCT can be downloaded from https://github.com/ztpub/DCT.

## CRediT author statement

**Jihong Hu:** Investigation, Methodology, Resources, Writing-original draft preparation. **Tao Zeng:** Methodology, Software, Writing-original draft preparation. **Qiongmei Xia:** Investigation, Resources. Liyu Huang: Investigation, Validation. **Yesheng Zhang:** Validation. **Chuanchao Zhang:** Software. **Yan Zeng:** Methodology. **Hui Liu:** Resources. **Shilai Zhang:** Investigation. **Guangfu Huang:** Investigation. **Wenting Wan:** Investigation. **Yi Ding:** Validation. **Fengyi Hu:** Supervision, Validation. **Congdang Yang:** Resources, Investigation. **Luonan Chen:** Conceptualization, Methodology, Software, Writing-reviewing and editing. **Wen Wang:** Conceptualization, Methodology, Supervision, Writing-reviewing and editing. All authors read and approved the final manuscript.

## Competing interests

The authors have declared no competing interests.

## Supplementary data

Supplementary Table S1 **The average temperature, average temperature difference, average relative humidity and monthly rainfall during the growth period of rice at Taoyuan and Jinghong (Xia et al., 2016)**.

Supplementary Table S2 **Differentially expressed genes between Taoyuan and Jinghong in different tissues using traditional expression analysis**.

Supplementary Table S3 **Detail information of the 26 genes which were reported to be associated with rice yield used in this study**.

Supplementary Table S4 **Top30 candidate genes in different tissues of rice variety 9311 screened by dynamic cross-tissue analysis**.

Supplementary Table S5 **Expression patterns of the 24 high yield candidate genes screened by dynamic cross-tissue analysis in rice variety 9311**.

Supplementary Table S6 **Candidate transcription factors regulating the yield of rice screened by dynamic cross-tissue analysis**.

Supplementary Table S7 **GO enrichment analysis of the top 30 candidate genes in different tissues of rice variety 9311**.

Supplementary Table S8 **Detail information of the primers used in this study**.

Supplementary File S1 **Algorithm of jcNMF**.

Supplementary File S2 **Convergence proof of the jcNMF algorithm**.

Supplementary Figure S3 **The differential co-expression network between the high-ranked top 30 genes in seven tissues and reported yield-associated genes.** DENs of tiller bud **(A)**, tiller root **(B)**, young panicle **(C)**, booting panicle **(D)**, booting root **(E)**, booting leaf **(F)** and flag leaf **(G)**.

Supplementary Figure S5 **GO enrichment analysis of the known yield-associated genes and candidate genes**. GO enrichment of the 26 known yield-associated genes (See Table S3) **(A)**. GO enrichment of the top 30 candidate genes in the young panicle analyzed by dynamic cross-tissue analysis **(B)**.
