# Supplementary material for: Identification of Key Genes for the Ultrahigh Yield of Rice Using Dynamic Cross-tissue Network Analysis
Source: Genomics Proteomics Bioinformatics. 2020 Jul 28;18(3):256–70. doi: 10.1016/j.gpb.2019.11.007 (PMC7801251; doi:10.1016/j.gpb.2019.11.007)
Supplement: Supplementary Table S1 — The average temperature, average temperature difference, average relative humidity and monthly rainfall during the growth period of rice at Taoyuan and Jinghong (Xia et al., 2016). [file mmc1.doc]

**Table S1** **The average temperature, average temperature difference, average relative humidity and monthly rainfall during the growth period of rice in Taoyuan and Jinghong (Xia et al., 2016)**

| **Time** | |  | **Average temperature ( °C )** | |  | **Average temperature difference ( °C )** | |  | **Average relative humidity ( % )** | | |  | **Monthly rainfall ( mm )** | |
| --- | --- | --- | --- | --- | --- | --- | --- | --- | --- | --- | --- | --- | --- | --- |
| **Taoyuan** | **Jinghong** |  | **Taoyuan** | **Jinghong** |  | **Taoyuan** | **Jinghong** |  | **Taoyuan** | | **Jinghong** |  | **Taoyuan** | **Jinghong** |
| May | March |  | 27.05 | 21.91 |  | 13.48 | 19.16 |  | 42.43 | | 58.90 |  | 21.40 | 2.00 |
| June | April |  | 28.17 | 23.55 |  | 12.22 | 13.07 |  | 51.36 | | 74.03 |  | 41.10 | 174.80 |
| July | May |  | 26.66 | 25.56 |  | 10.93 | 10.65 |  | 63.75 | | 74.13 |  | 199.30 | 121.60 |
| August | June |  | 25.76 | 27.19 |  | 10.97 | 10.13 |  | 68.38 | | 75.03 |  | 78.70 | 79.70 |
| Transplant-Heading | |  | 27.29 | 24.05 |  | 12.21 | 13.64 |  | 52.51 |  | 70.36 |  | 261.80 | 360.80 |
| Heading-Mature | |  | 25.76 | 27.41 |  | 10.97 | 9.56 |  | 68.38 |  | 75.03 |  | 78.70 | 74.90 |

*Note*: Xia QM, Li GY, Deng AF, Peng XY, Bai YL, Long RP, Yang CD. Studies on mechanism of high yield rice in special eco-site of Yunnan Province. Southwest China Journal of Agricultural Science 2016;29:6–10.
