# Supplementary material for: Identification of Key Genes for the Ultrahigh Yield of Rice Using Dynamic Cross-tissue Network Analysis
Source: Genomics Proteomics Bioinformatics. 2020 Jul 28;18(3):256–70. doi: 10.1016/j.gpb.2019.11.007 (PMC7801251; doi:10.1016/j.gpb.2019.11.007)
Supplement: Supplementary Table S3 — Detail information of the 26 genes which were reported to be associated with rice yield used in this study. [file mmc3.docx]

**Table S3 Detail information of 26 genes which were reported to associate with rice yield used in this study**

| ID | Gene | Annotation | References |
| --- | --- | --- | --- |
| LOC_Os08g41940 | *OsSPL16* | SOUAMOSA PROMOTER BINDING PROTEIN-LIKE 16 | Wang et al. 2012 |
| LOC_Os07g15770 | *Ghd7* | A CCT domain protein | Xue et al. 2008 |
| LOC_Os04g33740 | *GIF1* | GRAIN INCOMPLETE FILLING 1 | Wang et al. 2008 |
| LOC_Os07g05900 | *PROG1* | PROSTRATE GROWTH 1 | Jin et al. 2008 |
| LOC_Os08g39890 | *OsSPL14* | SOUAMOSA PROMOTER BINDING PROTEIN-LIKE 14 | Jiao et al. 2010; Miura et al. 2010 |
| LOC_Os02g14720 | *GW2* | RING-type protein with E3 ubiquitin ligase | Song et al.2007 |
| LOC_OS02g49410 | *Ghd8* | OsHAP3 subunit of a CCAAT-box binding protein (HAP complex) | Wei et al. 2010; Yan et al. 2011 |
| LOC_Os03g49880 | *OsTB1/FC1* | TCP transcription factor | Minakuchi et al. 2010 |
| LOC_Os06g06050 | *D3* | F-box LRR protein | Ishikawa et al., 2005 |
| LOC_Os04g46470 | *HTD1* | A family of CCD protein | Zou et al., 2006 |
| LOC_Os09g35980 | *TAC1* | Unknown protein | Yu et al., 2007 |
| LOC_Os11g37650 | *DWARF27* | Iron-containing protein | Lin et al., 2009 |
| LOC_Os06g40780 | *MOC1/SPA* | GRAS family nuclear protein | Li et al., 2003 |
| LOC_Os11g05470 | *RCN1* | Rice TFL1/CEN homolog | Nakagawa et al., 2002 |
| LOC_Os02g32950 | *RCN2* | Rice TFL1/CEN homolog | Nakashima et al., 2007 |
| LOC_Os01g61480 | *LAX1* | bHLH transcription factor | Komatsu et al., 2003 |
| LOC_Os01g10110 | *Gn1a* | Cytokinin oxidase | Ashikari et al., 2005 |
| LOC_Os06g45460 | *APO1* | F-box protein | Ikeda et al., 2007 |
| LOC_Os01g40630 | *LOG* | Cytokinin-activating enzyme | Kurakawa et al., 2007 |
| LOC_Os04g51000 | *RFL* | LFY homolog | Rao et al., 2008 |
| LOC_Os11g12740 | *SP1* | PTR family transporter | Li et al. 2009 |
| LOC_Os09g26999 | *DEP1* | PEBP like domain protein | Huang et al. 2009 |
| LOC_Os02g15950 | *EP3* | F-box protein | Piao et al. 2009 |
| LOC_Os02g05880 | *LRK1* | LRR receptor-like kinase | Zha et al. 2009 |
| LOC_Os07g42410 | *DEP2* | Unknown protein | Li et al. 2010 |
| LOC_Os02g15350 | *RPBF* | Prolamin box binding factor | Kawakatsu et al. 2009 |
