# Supplementary material for: Identification of Key Genes for the Ultrahigh Yield of Rice Using Dynamic Cross-tissue Network Analysis
Source: Genomics Proteomics Bioinformatics. 2020 Jul 28;18(3):256–70. doi: 10.1016/j.gpb.2019.11.007 (PMC7801251; doi:10.1016/j.gpb.2019.11.007)
Supplement: Supplementary Table S6 — Candidate transcription factors regulating the yield of rice screened by dynamic cross-tissue analysis. [file mmc6.docx]

**Table S6 Candidate transcription factors regulating the yield of rice screened by dynamic cross-tissue analysis**

| **ID** | **Gene** | **Annotation** |
| --- | --- | --- |
| LOC_Os07g03220 | *bZIP* | bZIP transcription factor domain containing protein |
| LOC_Os06g04850 |  | homeobox associated leucine zipper |
| LOC_Os01g53260 | *WRKY23* | WRKY23 |
| LOC_Os04g38400 |  | ethylene-insensitive 3 |
| LOC_Os04g32790 | *AP2* | AP2 domain containing protein |
| LOC_Os03g15790 |  | ZOS3-08 - C2H2 zinc finger protein |
| LOC_Os10g27380 |  | A family of CCD protein |
| LOC_Os05g02730 |  | homeobox domain containing protein |
| LOC_Os03g55164 | *WRKY4* | WRKY4 |
| LOC_Os01g58420 | *AP2* | AP2 domain containing protein |
| LOC_Os06g01620 |  | scarecrow |
| LOC_Os01g63980 |  | ZOS1-17 - C2H2 zinc finger protein |
| LOC_Os12g06080 |  | Cytokinin oxidase |
| LOC_Os04g43910 | *ARF* | auxin response factor |
| LOC_Os03g15660 | *AP2* | AP2 domain containing protein |
| LOC_Os06g14670 |  | ODORANT1 |
| LOC_Os01g48446 |  | no apical meristem protein |
| LOC_Os02g02820 |  | helix-loop-helix DNA-binding domain containing protein |
| LOC_Os09g35030 |  | dehydration-responsive element-binding protein |
| LOC_Os02g40070 | *AP2* | AP2-like ethylene-responsive transcription factor |
| LOC_Os03g12940 | *OsFBO13* | OsFBO13 - F-box and other domain containing protein |
| LOC_Os05g04740 |  | helix-loop-helix DNA-binding domain containing protein |
| LOC_Os08g36700 |  | HSF-type DNA-binding domain containing protein |
| LOC_Os05g10620 |  | no apical meristem protein |
| LOC_Os12g41880 | *NF-Y* | nuclear transcription factor Y subunit |
| LOC_Os05g06340 |  | GATA zinc finger domain containing protein |
| LOC_Os02g49880 |  | CCT/B-box zinc finger protein |
| LOC_Os10g41460 |  | transcription factor like protein |
| LOC_Os04g51560 | *WRKY68* | WRKY68 |
| LOC_Os12g06200 |  | E2F family transcription factor protein |
| LOC_Os06g15330 |  | CCT/B-box zinc finger protein |
| LOC_Os02g10860 | *bZIP* | bZIP transcription factor domain containing protein |
| LOC_Os05g50900 |  | helix-loop-helix DNA-binding protein, |
| LOC_Os02g51090 |  | RWP-RK domain-containing protein |
| LOC_Os03g56580 |  | no apical meristem protein |
| LOC_Os12g43790 | *bZIP* | bZIP transcription factor domain containing protein |
| LOC_Os01g59640 |  | no apical meristem protein |
| LOC_Os03g63750 |  | HSF-type DNA-binding domain containing protein |
| LOC_Os03g51330 |  | GRAS family transcription factor domain containing protein |
| LOC_Os07g47330 | *AP2* | AP2 domain containing protein |
| LOC_Os03g09170 | *ERF* | ethylene-responsive transcription factor |
| LOC_Os08g37580 |  | homeobox associated leucine zipper |
| LOC_Os06g46400 |  | ATCPSF30/CPSF30 |
| LOC_Os01g15640 |  | no apical meristem protein |
| LOC_Os09g35790 | *HSF* | HSF-type DNA-binding domain containing protein |
| LOC_Os11g04400 |  | GRAS family transcription factor containing protein |
| LOC_Os07g48560 |  | homeobox domain containing protein |
| LOC_Os08g06280 |  | LSD1 zinc finger domain containing protein |
| LOC_Os02g03960 |  | CPuORF1 - conserved peptide uORF-containing transcript |
| LOC_Os01g62460 |  | ZOS1-16 - C2H2 zinc finger protein |
| LOC_Os03g08500 | *AP2* | AP2 domain containing protein |
| LOC_Os08g06110 | *MYB* | MYB family transcription factor |
