# Supplementary material for: Identification of Key Genes for the Ultrahigh Yield of Rice Using Dynamic Cross-tissue Network Analysis
Source: Genomics Proteomics Bioinformatics. 2020 Jul 28;18(3):256–70. doi: 10.1016/j.gpb.2019.11.007 (PMC7801251; doi:10.1016/j.gpb.2019.11.007)
Supplement: Supplementary Table S8 — Detail information of the primers used in this study. [file mmc8.docx]

**Table S8 Detail information of the primers used in this study**

| **ID** | **Gene** | **Forward** | **Reverse** | **Propose** |
| --- | --- | --- | --- | --- |
| LOC_Os01g09620 | *Zinc finger* | CGCCTCCTCTTCCTCCTCCTC | ACTGCCGCTCATGGTCATCTG | qRT-PCR |
| LOC_Os09g24490 | *bHLH* | CTCCTACCCAACACCACCAAG | CGCATCGTCGTCGTCCTC | qRT-PCR |
| LOC_Os09g26420 | *AP2* | ATCAGCAAAGCACAAGAGGAAG | CCAAGCCAAACACGGACAC | qRT-PCR |
| LOC_Os06g11860 | *ERF* | CCTCGTCCTCGTCTTCTTCCTC | GGTGGTGGTGGTGGAGATGG | qRT-PCR |
| LOC_Os06g16400 | *HLH* | TGCCGTCGATTCCTCCTCCAAG | TGCTTCACCCCACCATCCTGTC | qRT-PCR |
| LOC_Os03g11614 | *OsMADS1* | AGCGGTTCTAGCACTGTTCTTG | CCTGGTGATGGTGAGCATGAG | qRT-PCR |
| LOC_Os05g41240 | *MYB* | CGCAGAGTACACAGAGTAACG | GAGCAATAGCATCCACAAAGC | qRT-PCR |
| LOC_Os02g44370 | *Scarecrow* | ATCTACCTCGCCCGTAACC | GCAGCCGTCGTCTTCATC | qRT-PCR |
| LOC_Os03g50885 | *Actin1* | ATCGCCCTGGACTATGAC | TATGAAGGAAGGCTGGAA | qRT-PCR |
|  | *OsSPL4* | GGCAGGTGAGGTGCCAGGTGGAA | AAACTTCCACCTGGCACCTCACC | Vector |
