# Supplementary material for: Identification of Key Genes for the Ultrahigh Yield of Rice Using Dynamic Cross-tissue Network Analysis
Source: Genomics Proteomics Bioinformatics. 2020 Jul 28;18(3):256–70. doi: 10.1016/j.gpb.2019.11.007 (PMC7801251; doi:10.1016/j.gpb.2019.11.007)
Supplement: Supplementary File S1 — Algorithm of jcNMF. [file mmc9.pdf]

File S1 Algorithm of jcNMF

Actually, the basic objective function can be abstracted as:

$$F\left(W^1_{M,K}, W^2_{M,K}, H^1_{K,S}, H^2_{K,S}\right) = \sum_{n=1,2} \left\| D^n_{M,S} - W^n_{M,K} H^n_{K,S} \right\|_F^2$$
$$+ \lambda_1 \left\| W^1_{M,K} (W^1_{M,K})^T - W^2_{M,K} (W^2_{M,K})^T \right\|_F^2$$
$$+ \lambda_2 \left[\sum_n \left\| W^n_{M,K} \right\|_F^2 + \left(\sum_j \left\| h^1_j \right\|_1^2 + \sum_j \left\| h^2_j \right\|_1^2 \right) \right]$$

Where M is the number of tissues, S is the number of genes, and K is the number of components from NMF. However, in this study, the rice seeds are the same which means they have similar genetic background. Thus it is reasonable to assume that the tissue-related matrices $W^1_{M,K}$ and $W^2_{M,K}$ would satisfy $W^1_{M,K}(W^1_{M,K})^T = W^2_{M,K}(W^2_{M,K})^T = R$ where R reflects the conserved correlations among tissues. The constraint R can also be further relaxed as the interactive matrix (A-B) indicating the similar samples from one tissue or stage, and the interactive matrix (C-D) indicating the similar samples from developing tissues or consecutive stages, where this constraint is also useful when original matrix has low-rank. Here, the data matrix D(M,S) represents the original input data (i.e. gene expression data); the matrices W and H represent the decomposed factor matrices respectively during NMF; the matrix h represents the residues; the matrices A and B together indicate the intra- similar matrix of tissues, and the matrices C and D together indicate the inter- similar matrix of tissues; the series of λ represent the penalty parameters.

Then, the objective function of jcNMF is formally defined for this study as:

$$F\left(W_{M,K}, H^1_{K,S}, H^2_{K,S}\right) = \sum_{n=1,2} \left\| D^n_{M,S} - W_{M,K} H^n_{K,S} \right\|_F^2$$
$$+ \lambda_1 \left\| W^T_{M,K} A - W^T_{M,K} B \right\|_F^2 + \lambda_2 \left\| W^T_{M,K} C - W^T_{M,K} D \right\|_F^2 \quad (1)$$
$$+ \lambda_3 \left[\left\| W_{M,K} \right\|_F^2 + \left(\sum_j \left\| h^1_j \right\|_1^2 + \sum_j \left\| h^2_j \right\|_1^2 \right) \right]$$

where $W_{M,K} \geq 0$, $H^n_{K,S} \geq 0$ and n = 1, 2; h^1_j and h^2_j are the j^1_{th} and j^2_{th} column of $H^1_{K,S}$ and $H^2_{K,S}$ respectively.

The algorithm of jcNMF shown in bellows is expected to minimalize such an objective function, i.e.

$$\min F\left(W_{M,K}, H^1_{K,S}, H^2_{K,S}\right) \quad (2)$$

According to the popular multiplicative updating algorithm developed for NMF (Lee and Seung, 1999), a variant is expanded to solve jcNMF. As well-known, the objective function of general NMFis convex in $W_{M,K}$ only or $H^n_{K,S}$ only, but it is not convex in both types of variables

together. Therefore, it is unrealistic to expect an algorithm to find the global minimum for NMF, and this is the same for jcNMF problem. Thus, the following multiplicative updating algorithm for jcNMF is actually to identify the local minimum of the objective function F as formula (2).

Step 1:

Initialize $W_{M,K}$,$H^1_{K,S}$, and $H^2_{K,S}$ with non-negative values, and set the iteration index t=0.

Step 2:

Fix $W_{M,K}$, and solve the constrained problem of $H^1_{K,S}$ and $H^2_{K,S}$

$$\min_{H} \sum_{n\in\{1,2\}} \left|\left|D^n_{M,S} - W_{M,K} \cdot H^n_{K,S}\right|\right|_F^2 + \lambda_3(\sum_j \left|\left|h^1_j\right|\right|_1^2 + \sum_j \left|\left|h^2_j\right|\right|_1^2)$$

That is, update $H^1_{K,S}$ and $H^2_{K,S}$ with

$$h^1_{ij} \leftarrow h^1_{ij} \frac{((W_{M,K})^T \cdot D^1_{M,S})_{ij}}{((W_{M,K})^T W_{M,K} H^1_{K,S} + \lambda_3 e_{k\times k} H^1_{K,S})_{ij}}$$

$$h^2_{ij} \leftarrow h^2_{ij} \frac{((W_{M,K})^T \cdot D^2_{M,S})_{ij}}{((W_{M,K})^T W_{M,K} H^2_{K,S} + \lambda_3 e_{k\times k} H^2_{K,S})_{ij}}$$

Step 3:

Fix $H^1_{K,S}$ and $H^2_{K,S}$, and solve the constrained problem of $W_{M,K}$

$$\min_{W} \sum_{n\in\{1,2\}} \left|\left|D^n_{M,S} - W_{M,K} \cdot H^n_{K,S}\right|\right|_F^2$$

$$+\lambda_1 \sum_{n\in\{1,2\}} \left|\left|(W_{M,K})^T \cdot A - (W_{M,K})^T \cdot B\right|\right|_F^2$$

$$+\lambda_2 \sum_{n\in\{1,2\}} \left|\left|(W_{M,K})^T \cdot C - (W_{M,K})^T \cdot D\right|\right|_F^2$$

$$+\lambda_3 ||W_{M,K}||_F^2$$

Then, update $W_{M,K}$ with

$$w_{ij} \leftarrow w_{ij} \frac{(D^1_{M,S} \cdot (H^1_{K,S})^T + D^2_{M,S} \cdot (H^2_{K,S})^T + \lambda_1(AB^T W_{M,K} + BA^T W_{M,K}) + \lambda_2(CD^T W_{M,K} + DC^T W_{M,K}))_{ij}}{(W_{M,K} H^1_{K,S}(H^1_{K,S})^T + W_{M,K} H^2_{K,S}(H^2_{K,S})^T + \lambda_1(AA^T W_{M,K} + BB^T W_{M,K}) + \lambda_2(CC^T W_{M,K} + DD^T W_{M,K}) + \lambda_3 W_{M,K})_{ij}}$$

Step 4:

Let t < −t+1, and repeat Steps2−3 until convergence criteria are satisfied.
