# Supplementary material for: Identification of Key Genes for the Ultrahigh Yield of Rice Using Dynamic Cross-tissue Network Analysis
Source: Genomics Proteomics Bioinformatics. 2020 Jul 28;18(3):256–70. doi: 10.1016/j.gpb.2019.11.007 (PMC7801251; doi:10.1016/j.gpb.2019.11.007)
Supplement: Supplementary File S2 — Convergence proof of the jcNMF algorithm. [file mmc10.pdf]

File S2 Convergence proof of the algorithm of jcNMF

Firstly, according to the linear algebra, the objective function F can be reformulated as follows:

$$\begin{aligned} F = & \sum_{n=1}^{2}\left[Tr\left(D_{M,S}^{n}\left(D_{M,S}^{n}\right)^{T}\right)+Tr\left(W_{M,K}H_{K,S}^{n}\left(H_{K,S}^{n}\right)^{T}W_{M,K}^{T}\right)-2Tr\left(D_{M,S}^{n}\left(H_{K,S}^{n}\right)^{T}W_{M,K}^{T}\right)\right] \\ & +\lambda_{1}\left[Tr\left(W_{M,K}^{T}AA^{T}W_{M,K}\right)+Tr\left(W_{M,K}^{T}BB^{T}W_{M,K}\right)-Tr\left(W_{M,K}^{T}AB^{T}W_{M,K}\right)-Tr\left(W_{M,K}^{T}BA^{T}W_{M,K}\right)\right] \\ & +\lambda_{2}\left[Tr\left(W_{M,K}^{T}CC^{T}W_{M,K}\right)+Tr\left(W_{M,K}^{T}DD^{T}W_{M,K}\right)-Tr\left(W_{M,K}^{T}CD^{T}W_{M,K}\right)-Tr\left(W_{M,K}^{T}DC^{T}W_{M,K}\right)\right] \\ & +\lambda_{3}\left[Tr\left(W_{M,K}W_{M,K}^{T}\right)+\sum_{I=1}^{2}e_{1\times k}H_{K,S}^{n}\left(H_{K,S}^{n}\right)^{T}e_{1\times k}^{T}\right] \end{aligned} \qquad (3)$$

Let φ_{ij} and ϕ_{ij}^{I} be the Lagrange multipliers for the constraints $\left(W_{M,K}\right)_{ij}\geq 0$ and $\left(H_{K,S}^{n}\right)_{ij}\geq 0$ respectively. The Lagrange L is

$$L\left(W,H_{I}\right)=F+Tr\left(\varphi W_{M,K}^{T}\right)+\sum_{n=1}^{2}Tr\left(\phi_{n}\left(H_{K,S}^{n}\right)^{T}\right)$$

where $\Psi=\left[\varphi_{ij}\right]$ and $\Phi_{n}=\left[\phi_{ij}^{n}\right]$. The partial derivatives of L with respect to $W_{M,K}$ and $H_{K,S}^{n}$ are:

$$\frac{\partial L}{\partial H_{K,S}^{1}}=-2W_{M,K}^{T}D_{M,S}^{1}+2W_{M,K}^{T}W_{M,K}H_{K,S}^{1}+\lambda_{3}2e_{K\times K}H_{K,S}^{1}+\Phi_{1}$$

$$\frac{\partial L}{\partial H_{K,S}^{2}}=-2W_{M,K}^{T}D_{M,S}^{2}+2W_{M,K}^{T}W_{M,K}H_{K,S}^{2}+\lambda_{3}2e_{K\times K}H_{K,S}^{2}+\Phi_{2}$$

$$\begin{aligned} \frac{\partial L}{\partial W_{M,K}}= & \sum_{n=1}^{2}\left[-2D_{M,S}^{n}\left(H_{K,S}^{n}\right)^{T}+2W_{M,K}H_{K,S}^{n}\left(H_{K,S}^{n}\right)^{T}\right] \\ & +\lambda_{1}\left[2AA^{T}W_{M,K}+2BB^{T}W_{M,K}-2AB^{T}W_{M,K}-2BA^{T}W_{M,K}\right] \\ & +\lambda_{2}\left[2CC^{T}W_{M,K}+2DD^{T}W_{M,K}-2CD^{T}W_{M,K}-2DC^{T}W_{M,K}\right] \\ & +\lambda_{3}2W_{M,K}+\Psi \end{aligned}$$

Based on the KKT conditions $\varphi_{ij}\left(W_{M,K}\right)_{ij}=0$ and $\phi_{ij}^{I}\left(H_{K,S}^{n}\right)_{ij}=0$, we get the following equations for $\left(W_{M,K}\right)_{ij}$, $\left(H_{K,S}^{1}\right)_{ij}$ and $\left(H_{K,S}^{2}\right)_{ij}$ respectively:

$$\left(-2W_{M,K}^{T}D_{M,S}^{1}\right)_{ij}\left(H_{K,S}^{1}\right)_{ij}+\left(2W_{M,K}^{T}W_{M,K}H_{K,S}^{1}+\lambda_{3}2e_{k\times k}H_{K,S}^{1}\right)_{ij}\left(H_{K,S}^{1}\right)_{ij}=0$$

$$\left(-2W_{M,K}^{T}D_{M,S}^{2}\right)_{ij}\left(H_{K,S}^{2}\right)_{ij}+\left(2W_{M,K}^{T}W_{M,K}H_{K,S}^{2}+\lambda_{3}2e_{k\times k}H_{K,S}^{2}\right)_{ij}\left(H_{K,S}^{2}\right)_{ij}=0$$

$$\begin{aligned} & \left(-2D_{M,S}^{1}\left(H_{K,S}^{1}\right)^{T}-2D_{M,S}^{2}\left(H_{K,S}^{2}\right)^{T}-\lambda_{1}2AB^{T}W_{M,K}-\lambda_{1}2BA^{T}W_{M,K}-\lambda_{2}2CD^{T}W_{M,K}-\lambda_{2}2DC^{T}W_{M,K}\right)_{ij}\left(W_{M,K}\right)_{ij} \\ & +\left(2W_{M,K}H_{K,S}^{1}\left(H_{K,S}^{1}\right)^{T}+2W_{M,K}H_{K,S}^{2}\left(H_{K,S}^{2}\right)^{T}+\lambda_{1}2AA^{T}W_{M,K}+\lambda_{1}2BB^{T}W_{M,K}+\lambda_{2}2CC^{T}W_{M,K}+\lambda_{2}DD^{T}W_{M,K}+\lambda_{3}2W_{M,K}\right)_{ij}\left(W_{M,K}\right)_{ij}=0 \end{aligned}$$

Then we can get the following updating rules:

$$\left(h_{K,S}^{1}\right)_{ij} \leftarrow \left(h_{K,S}^{1}\right)_{ij} \cdot \frac{\left(W_{M,K}^{T} D_{M,S}^{1}\right)_{ij}}{\left(W_{M,K}^{T} W_{M,K} H_{K,S}^{1} + \lambda_3 e_{K\times K} H_{K,S}^{1}\right)_{ij}}$$

$$\left(h_{K,S}^{2}\right)_{ij} \leftarrow \left(h_{K,S}^{2}\right)_{ij} \cdot \frac{\left(W_{M,K}^{T} D_{M,S}^{2}\right)_{ij}}{\left(W_{M,K}^{T} W_{M,K} H_{K,S}^{2} + \lambda_3 e_{K\times K} H_{K,S}^{2}\right)_{ij}}$$

$$\left(w_{M,K}\right)_{ij} \leftarrow \left(w_{M,K}\right)_{ij} \cdot \frac{\left[D_{M,S}^{1}\left(H_{K,S}^{1}\right)^{T} + D_{M,S}^{2}\left(H_{K,S}^{2}\right)^{T} + \lambda_1\left(AB^{T}W_{M,K} + BA^{T}W_{M,K}\right) + \lambda_2\left(CD^{T}W_{M,K} + DC^{T}W_{M,K}\right)\right]_{ij}}{\left[W_{M,K}H_{K,S}^{1}\left(H_{K,S}^{1}\right)^{T} + W_{M,K}H_{K,S}^{2}\left(H_{K,S}^{2}\right)^{T} + \lambda_1\left(AA^{T}W_{M,K} + BB^{T}W_{M,K}\right) + \lambda_2\left(CC^{T}W_{M,K} + DD^{T}W_{M,K}\right) + \lambda_3 W_{M,K}\right]_{ij}}$$

Secondly, the convergence of the above updating rules to a local optimum is guaranteed by the following theorem.

Theorem 1 The objective function F of the jcNMF problem is non-increasing under the correspondingupdating rules above. The objective function is finite and invariant under these updating rules if and only if $W_{M,K}$, $H_{K,S}^{1}$ and $H_{K,S}^{2}$ are at a stationary point.

Actually, the theorem 1 can be proved by expanding the principle of convergence proof of general NMF. Different from conventional NMF, the objective function F of jcNMF could be unbounded. Only when the objective is finite, the jcNMF can get a local stable solution, and thus, it is necessary to show that F is non-increasing under the corresponding updating rules. In particular, here we prove that the F is non-increasing under the updating rule for *W*, and the same feature under the updating rule for $H_{K,S}^{1}$ and $H_{K,S}^{2}$ can be similarly proved. We will adopt the same strategy used by Lee and Seung (2001) that introduced an auxiliary function in the Expectation-Maximization algorithm.

Definition 1 $G\left(w_{M,K}, w_{M,K}^{'}\right)$ is an auxiliary function for $F\left(w_{M,K}\right)$, where the conditions $G\left(w_{M,K}, w_{M,K}^{'}\right) \geq F\left(w_{M,K}\right)$ and $G\left(w_{M,K}, w_{M,K}\right) \geq F\left(w_{M,K}\right)$ should be satisfied.

Due to the following property, this auxiliary function will be very useful for the following proof.

Lemma 1 If G is an auxiliary function of F, then F is non-increasing under the updating rule as $w_{M,K}^{(t+1)} = \arg\min G\left(w_{M,K}, w_{M,K}^{(t)}\right)$.

With a proper auxiliary function, the updating rule for W is exactly the update in this Lemma.

Taking into account any element $\left(W_{M,K}\right)_{ab}$ in W, we use the F_{ab} to denote the part of F that is only relevant to $\left(W_{M,K}\right)_{ab}$. It is easy to see that

$$\begin{aligned} F_{ab}^{'} = \left(\frac{\partial L}{\partial W_{M,K}}\right)_{ab} &= \{ \sum_{n=1}^{2}\left[-2D_{M,S}^{n}\left(H_{K,S}^{n}\right)^{T} + 2W_{M,K}H_{K,S}^{n}\left(H_{K,S}^{n}\right)^{T}\right] \\ &+ \lambda_1\left[2AA^{T}W_{M,K} + 2BB^{T}W_{M,K} - 2AB^{T}W_{M,K} - 2BA^{T}W_{M,K}\right] \\ &+ \lambda_2\left[2CC^{T}W_{M,K} + 2DD^{T}W_{M,K} - 2CD^{T}W_{M,K} - 2DC^{T}W_{M,K}\right] + \lambda_3 2W_{M,K} \}_{ab} \end{aligned}$$

$$F''_{ab} = \left(\sum_{n=1}^{2} 2H_{K,S}^{n} \left(H_{K,S}^{n} \right)^{T} \right)_{bb} + \left[2\lambda_1 (A-B)(A-B)^T + 2\lambda_2 (C-D)(C-D)^T + 2\lambda_3 \right]_{aa}$$

Lemma 2 An auxiliary function for F_{ab} can be:

$$G\left(w_{M,K}, \left(W_{M,K}^{(t)}\right)_{ab}\right) = F_{ab}\left(\left(W_{M,K}^{(t)}\right)_{ab}\right) + F'_{ab}\left(\left(W_{M,K}^{(t)}\right)_{ab}\right)\left(w_{M,K} - \left(W_{M,K}^{(t)}\right)_{ab}\right) + K_{ab}\left(W_{M,K}^{(t)}\right)\left(w_{M,K} - \left(W_{M,K}^{(t)}\right)_{ab}\right)^2$$

where:

$$K\left(W_{M,K}\right) = \frac{W_{M,K}\left(\sum_{n=1}^{2} H_{K,S}^{n}\left(H_{K,S}^{n}\right)^{T}\right) + \left[2\lambda_1\left(AA^T + BB^T\right) + 2\lambda_2\left(CC^T + DD^T\right) + 2\lambda_3\right]W_{M,K}}{W_{M,K}}$$

$$K_{ab}\left(W_{M,K}^{(t)}\right) = \frac{\left\{W_{M,K}^{(t)}\left(\sum_{\mathrm{n}=1}^{2}\left(H_{K,S}^{n}\right)\left(H_{K,S}^{n}\right)^{T}\right) + \left[2\lambda_1\left(AA^T + BB^T\right) + 2\lambda_2\left(CC^T + DD^T\right) + 2\lambda_3\right]W_{M,K}^{(t)}\right\}_{ab}}{\left(W_{M,K}^{(t)}\right)_{ab}}$$

Proof: Obviously, $G(w,w) = F_{ab}(w)$. Here we only show that:

$$G\left(w_{M,K}, \left(w_{M,K}^{(t)}\right)_{ab}\right) \geq F_{ab}\left(w_{M,K}\right)$$

To achieve this, we compare the Taylor series expansion of $F_{ab}\left(w_{M,K}\right)$

$$\begin{aligned} F_{ab}\left(w_{M,K}\right) &= F_{ab}\left(\left(W_{M,K}^{(t)}\right)_{ab}\right) \\ &\quad + F'_{ab}\left(\left(W_{M,K}^{(t)}\right)_{ab}\right)\left(w_{M,K} - \left(W_{M,K}^{(t)}\right)_{ab}\right) \\ &\quad + \left\{\left(\sum_{n=1}^{2} H_{K,S}^{n}\left(H_{K,S}^{n}\right)^{T}\right)_{bb} + \left[2\lambda_1 (A-B)(A-B)^T + 2\lambda_2 (C-D)(C-D)^T + 2\lambda_3\right]_{aa}\right\}\left(w_{M,K} - \left(W_{M,K}^{(t)}\right)_{ab}\right)^2 \end{aligned}$$

with the auxiliary function $G\left(w_{M,K}, \left(w_{M,K}^{(t)}\right)_{ab}\right)$ to find that $G\left(w_{M,K}, \left(w_{M,K}^{(t)}\right)_{ab}\right) \geq F_{ab}\left(w_{M,K}\right)$ is equivalent to

$$\frac{\left\{W_{M,K}^{(t)}\left(\sum_{n=1}^{2} H_{K,S}^{n}\left(H_{K,S}^{n}\right)^{T}\right) + \left[2\lambda_1\left(AA^T + BB^T\right) + 2\lambda_2\left(CC^T + DD^T\right) + 2\lambda_3\right]W_{M,K}^{(t)}\right\}_{ab}}{\left(W_{M,K}^{(t)}\right)_{ab}} \geq \left(\sum_{n=1}^{2} H_{K,S}^{n}\left(H_{K,S}^{n}\right)^{T}\right)_{bb} + \left[2\lambda_1 (A-B)(A-B)^T + 2\lambda_2 (C-D)(C-D)^T + 2\lambda_3\right]_{aa}$$

Obviously, we have

$$
\begin{aligned}
&\left\{W_{M,K}^{(t)}\left(\sum_{n=1}^{2}H_{K,S}^{n}\left(H_{K,S}^{n}\right)^{T}\right)+\left[2\lambda_1\left(AA^T+BB^T\right)+2\lambda_2\left(CC^T+DD^T\right)+2\lambda_3\right]W_{M,K}^{(t)}\right\}_{ab}\\
&=\sum_k\left\{\left(W_{M,K}^{(t)}\right)_{ak}\left(\sum_{n=1}^{2}H_{K,S}^{n}\left(H_{K,S}^{n}\right)^{T}\right)_{kb}+\left[2\lambda_1\left(AA^T+BB^T\right)+2\lambda_2\left(CC^T+DD^T\right)+2\lambda_3\right]_{ak}\left(W_{M,K}^{(t)}\right)_{kb}\right\}\\
&\geq\left(W_{M,K}^{(t)}\right)_{ab}\left(\sum_{n=1}^{2}H_{K,S}^{n}\left(H_{K,S}^{n}\right)^{T}\right)_{bb}+\left[2\lambda_1(A-B)(A-B)^T+2\lambda_2(C-D)(C-D)^T+2\lambda_3\right]_{aa}\left(W_{M,K}^{(t)}\right)_{ab}\\
&=\left(W_{M,K}^{(t)}\right)_{ab}\left\{\left(\sum_{n=1}^{2}H_{K,S}^{n}\left(H_{K,S}^{n}\right)^{T}\right)_{bb}\left[2\lambda_1(A-B)(A-B)^T+2\lambda_2(C-D)(C-D)^T+2\lambda_3\right]_{aa}\right\}
\end{aligned}
\quad\square
$$

Proof of Theorem 1

$$
\begin{aligned}
\left(W_{M,K}^{(t+1)}\right)_{ab}&=\left(W_{M,K}^{(t)}\right)_{ab}-\left(W_{M,K}^{(t)}\right)_{ab}\frac{F_{ab}^{'}\left(\left(W_{M,K}^{(t)}\right)_{ab}\right)}{\left(\sum_{n=1}^{2}2W_{M,K}H_{K,S}^{n}\left(H_{K,S}^{n}\right)^{T}+2\lambda_1\left(AA^T+BB^T\right)W_{M,K}+2\lambda_2\left(CC^T+DD^T\right)W_{M,K}+2\lambda_3W_{M,K}\right)_{ab}}\\
&=\left(W_{M,K}^{(t)}\right)_{ab}\frac{\left[D_{M,S}^{1}\left(H_{K,S}^{1}\right)^{T}+D_{M,S}^{2}\left(H_{K,S}^{2}\right)^{T}+\lambda_1\left(AB^TW_{M,K}+BA^TW_{M,K}\right)+\lambda_2\left(CD^TW_{M,K}+DC^TW_{M,K}\right)\right]_{ab}}{\left[W_{M,K}H_{K,S}^{1}\left(H_{K,S}^{1}\right)^{T}+W_{M,K}H_{K,S}^{2}\left(H_{K,S}^{2}\right)^{T}+\lambda_1\left(AA^TW_{M,K}+BB^TW_{M,K}\right)+\lambda_2\left(CC^TW_{M,K}+DD^TW_{M,K}\right)+\lambda_3W_{M,K}\right]_{ab}}
\end{aligned}
$$

We can get the following updating rule based on the auxiliary function $G\left(w_{M,K},\left(w_{M,K}^{(t)}\right)_{ab}\right)$

(4)

Due to the property of the auxiliary function $G\left(w_{M,K},\left(w_{M,K}^{(t)}\right)_{ab}\right)$ for F_{ab} , F_{ab} is non-increasing under this updating rule.
