# Supplementary material for: Identification of Key Genes for the Ultrahigh Yield of Rice Using Dynamic Cross-tissue Network Analysis
Source: Genomics Proteomics Bioinformatics. 2020 Jul 28;18(3):256–70. doi: 10.1016/j.gpb.2019.11.007 (PMC7801251; doi:10.1016/j.gpb.2019.11.007)

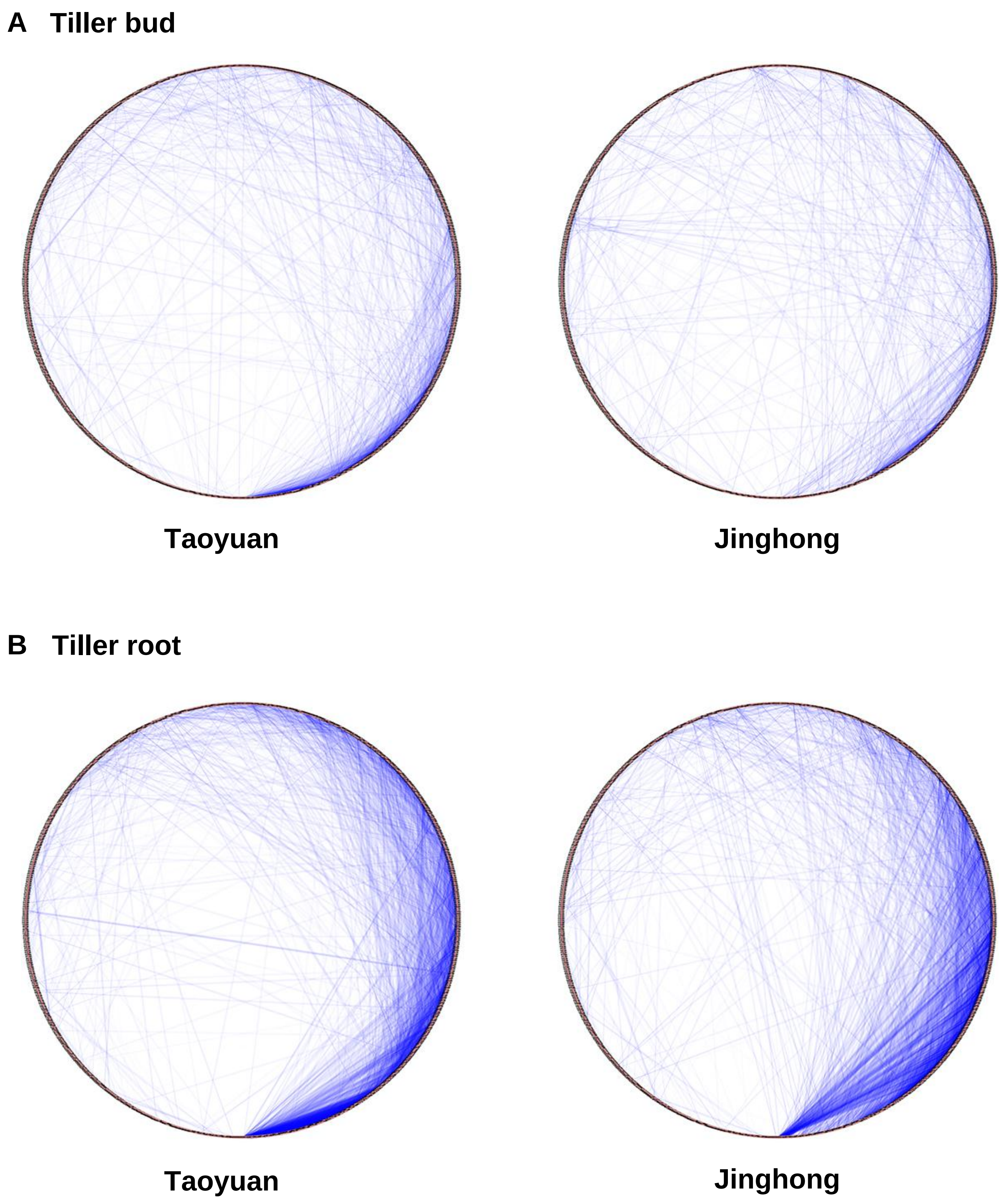
A Tiller bud
Taoyuan
Jinghong
B Tiller root
Taoyuan
Jinghong

C Young panicle

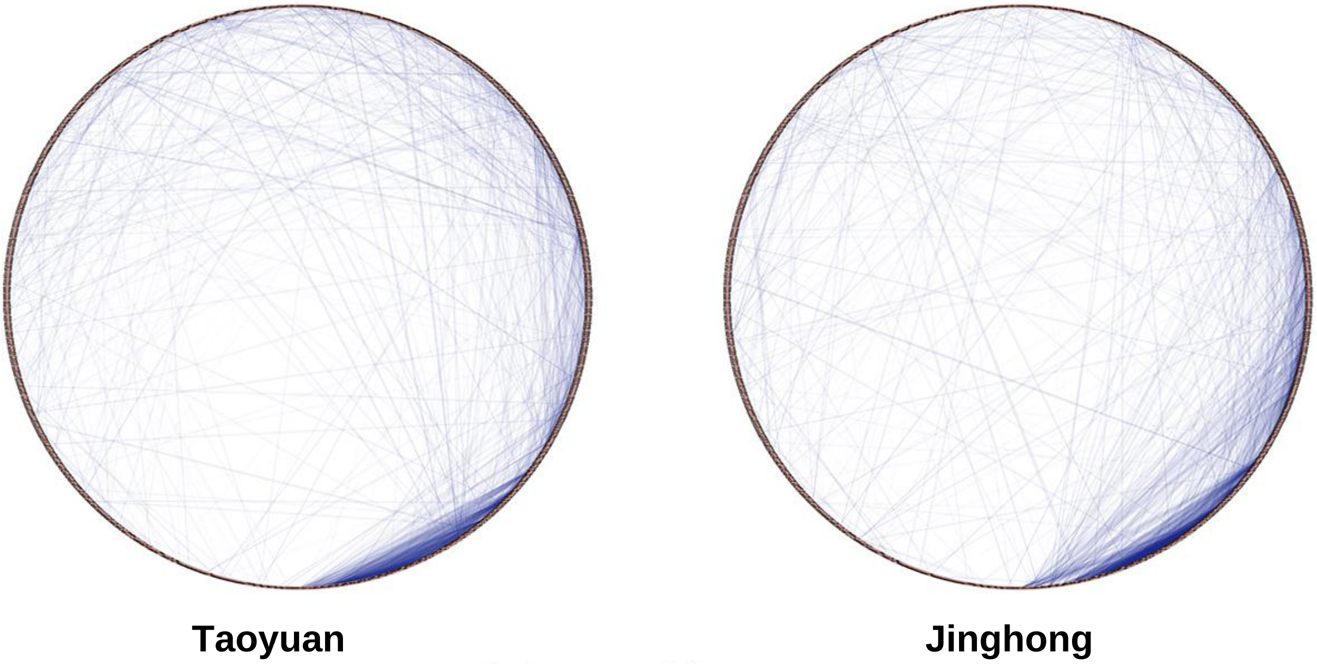

D Booting panicle

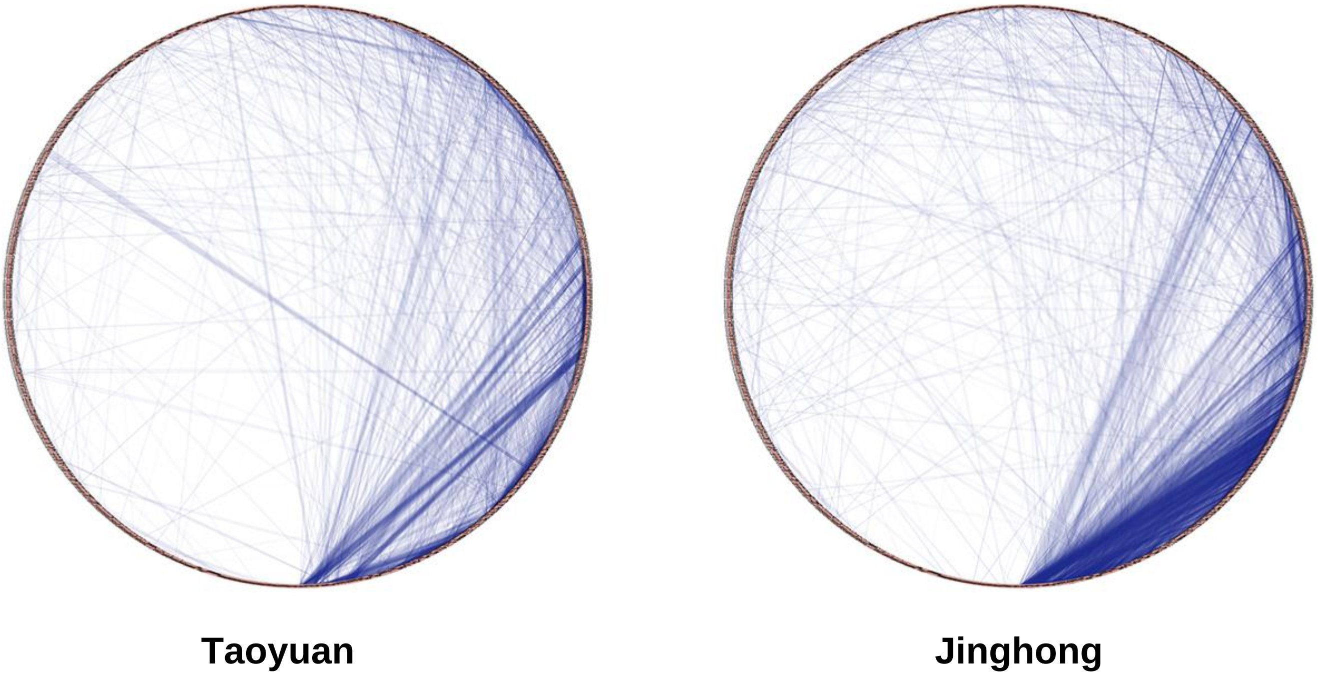

E Booting root

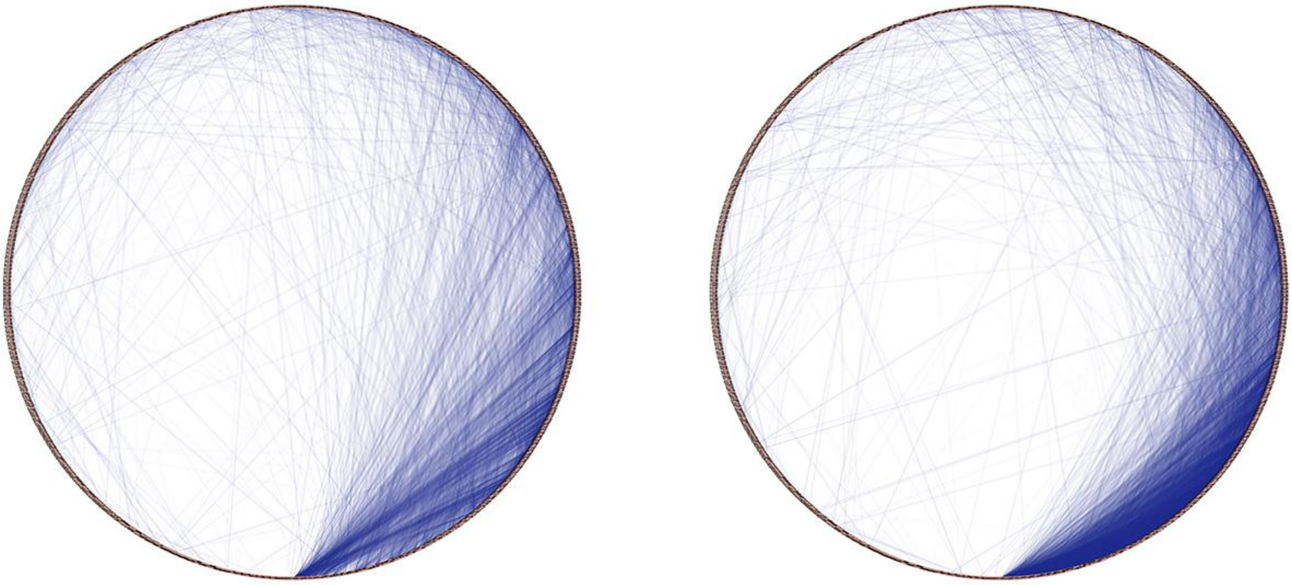

Taoyuan Jinghong

F Booting leaf

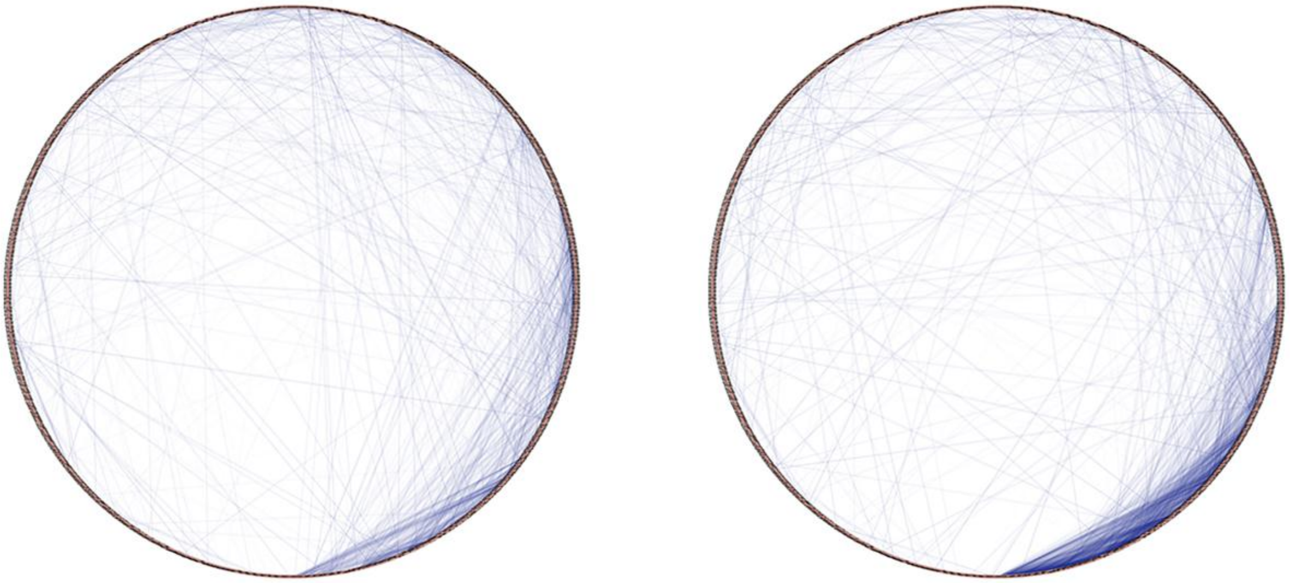

Taoyuan Jinghong

G Flag leaf

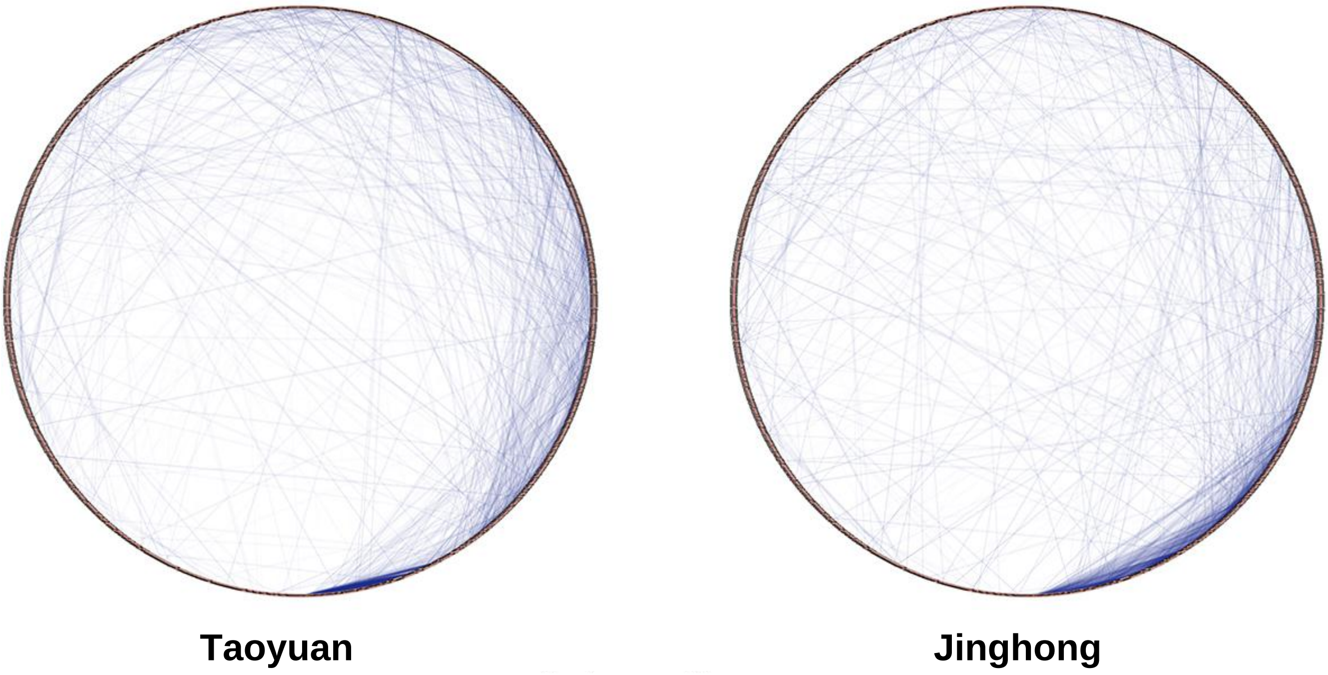

Supplement: Supplementary Figure S3 — The differential co-expression network between the high-ranked top 30 genes in seven tissues and reported yield-associated genes. DENs of tiller bud (A), tiller root (B), young panicle (C), booting panicle (D), booting root (E), booting leaf (F) and flag leaf (G). [file mmc11.pdf]
