# Supplementary figures and images for: Identification of Key Genes for the Ultrahigh Yield of Rice Using Dynamic Cross-tissue Network Analysis
Source: Genomics Proteomics Bioinformatics. 2020 Jul 28;18(3):256–70. doi: 10.1016/j.gpb.2019.11.007 (PMC7801251; doi:10.1016/j.gpb.2019.11.007)

A GO enrichment of the known yield genes

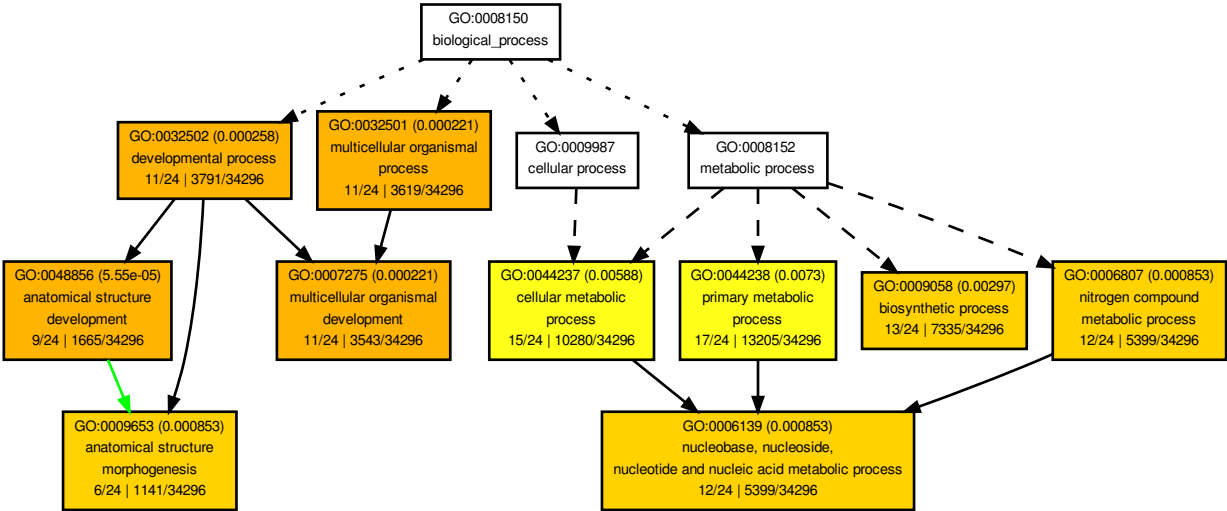

B GO enrichment of candidate yield genes

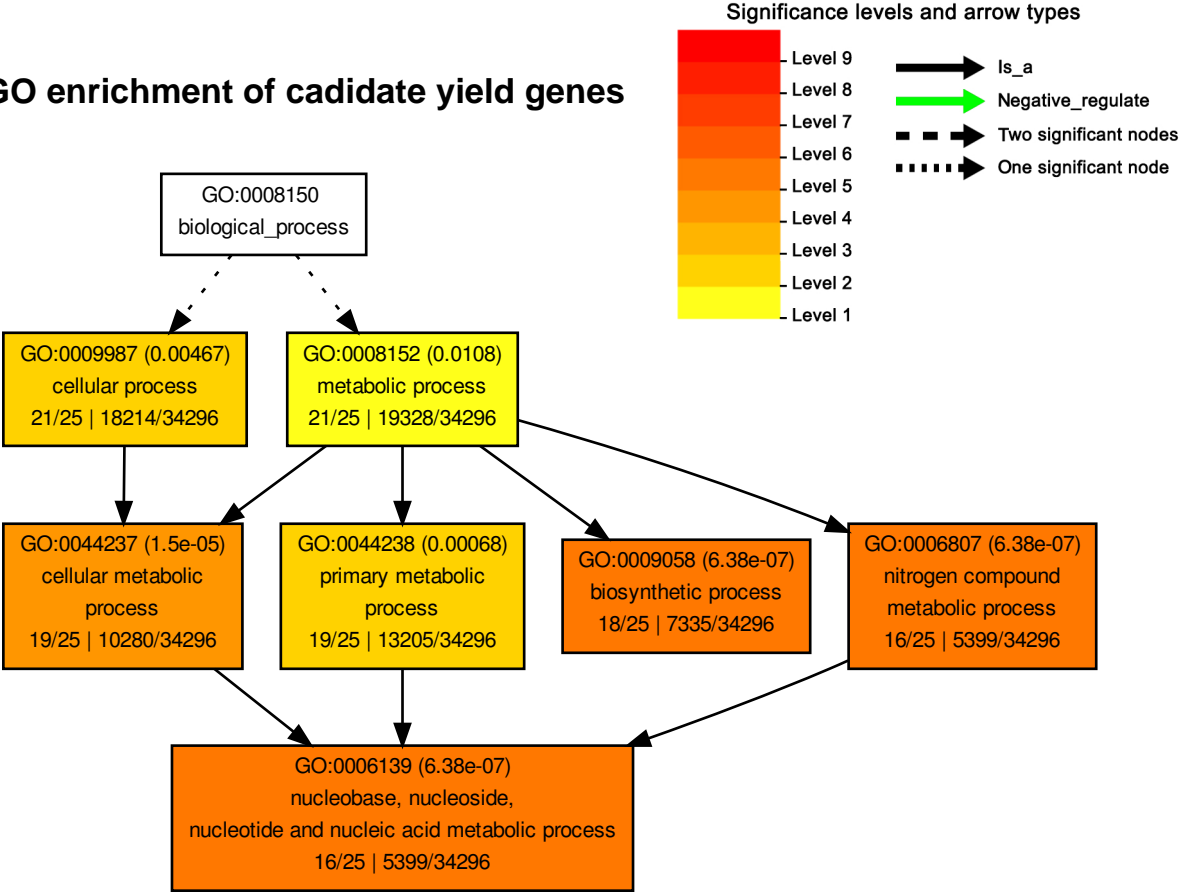

Supplement: Supplementary Figure S5 — GO enrichment analysis of the known yield-associated genes and candidate genes. GO enrichment of the 26 known yield-associated genes (See Table S3) (A). GO enrichment of the top 30 candidate genes in the young panicle analyzed by dynamic cross-tissue analysis (B). [file mmc12.pdf]
